# A genome-wide in vivo CRISPR screen identifies essential regulators of T cell migration to the CNS in a multiple sclerosis model

**DOI:** 10.1038/s41593-023-01432-2

**Published:** 2023-09-14

**Authors:** Arek Kendirli, Clara de la Rosa, Katrin F. Lämmle, Klara Eglseer, Isabel J. Bauer, Vladyslav Kavaka, Stephan Winklmeier, La Zhuo, Christian Wichmann, Lisa Ann Gerdes, Tania Kümpfel, Klaus Dornmair, Eduardo Beltrán, Martin Kerschensteiner, Naoto Kawakami

**Affiliations:** 1 Institute of Clinical Neuroimmunology, University Hospital, Ludwig-Maximilians-Universität München, Munich, Germany; 2 Biomedical Center (BMC), Faculty of Medicine, Ludwig-Maximilians-Universität München, Martinsried, Germany; 3 Graduate School of Systemic Neurosciences, Ludwig-Maximilians-Universität München, Munich, Germany; 4 Division of Transfusion Medicine, Cell Therapeutics and Haemostaseology, University Hospital, Ludwig-Maximilians-Universität München, Munich, Germany; 5 Munich Cluster for Systems Neurology (SyNergy), Munich, Germany

**Keywords:** Neuroimmunology, Blood-brain barrier, Autoimmunity, Multiple sclerosis, Lymphocytes

## Abstract

Multiple sclerosis (MS) involves the infiltration of autoreactive T cells into the CNS, yet we lack a comprehensive understanding of the signaling pathways that regulate this process. Here, we conducted a genome-wide in vivo CRISPR screen in a rat MS model and identified 5 essential brakes and 18 essential facilitators of T cell migration to the CNS. While the transcription factor ETS1 limits entry to the CNS by controlling T cell responsiveness, three functional modules, centered around the adhesion molecule α4-integrin, the chemokine receptor CXCR3 and the GRK2 kinase, are required for CNS migration of autoreactive CD4^+^ T cells. Single-cell analysis of T cells from individuals with MS confirmed that the expression of these essential regulators correlates with the propensity of CD4^+^ T cells to reach the CNS. Our data thus reveal key regulators of the fundamental step in the induction of MS lesions.

## Abstract (web summary)

Multiple sclerosis (MS) lesions are initiated by the infiltration of T cells to the CNS. Here, Kendirli et al. use a genome-wide CRISPR screen in an MS model to define the molecules that regulate T cell entry to the CNS.

## Main

MS is the most common disabling neurological disease in young adults. In MS, the cascade of tissue injury is initiated when activated autoreactive T cells infiltrate the CNS^1–3^. The importance of this step in MS pathogenesis is well evidenced from studies in rodent models of the disease and in humans. The capacity of CD4^+^ T cells to induce CNS inflammation has, for example, been demonstrated in rodent experimental autoimmune encephalomyelitis (EAE) models, in which activated T cells recognizing myelin basic protein (MBP) are transferred into naïve rodents where they induce an MS-like disease^4^. Experiments in such models have delineated the migratory path of encephalitogenic T cells to the CNS^5^; uncovered the compartments and cellular interactions that shape the induction of CNS inflammation^6,7^ and aided in the identification of adhesion molecules, such as α4-integrin, that are required for T cell migration to the CNS^6,8^. Clinical data have confirmed the importance of these processes in individuals with MS, showing that many of the gene loci conferring increased risk of the disease are predicted to affect CD4^+^ T cell activation and differentiation^9^; that there is an MS-associated immune gene signature in a subset of CD4^+^ T cells in monozygotic twins discordant for the disease^10^; that CD4^+^ T cells start colonizing the CNS from early stages of the disease^11^; and that therapies targeting T cell migration can be effective in ameliorating MS^12^.

Despite significant advances in our understanding of MS and how to treat it, most studies to date have focused on assessing and validating the roles of molecules known to be involved in T cell trafficking. However, this has left key knowledge gaps in the field, and we lack a comprehensive understanding of the essential molecular cues and signaling streams that enable or limit T cell entry to the CNS and may thus represent alternative targets for therapy. The advent of CRISPR gene editing technology now raises the possibility of conducting comprehensive and unbiased loss-of-function screens in disease models in vivo: indeed genome-wide CRISPR screens have been successfully used to answer questions related to cancer initiation, propagation and therapy^13,14^, as well as to reveal the mechanisms regulating critical immunological processes including T cell activation, proliferation and fate determination^15–17^. To date, this powerful approach has not been applied to study the initiation of CNS inflammation.

Here we used a rodent MS model and combined an unbiased genome-wide CRISPR screen with functional in vivo validation studies, multiphoton microscopy and in vitro mechanistic experiments, to provide a definite molecular characterization of the central step in MS pathogenesis, the infiltration of autoreactive T cells to the CNS.

## Results

### CRISPR screen identifies essential regulators of T cell migration to the CNS

**Fig. 1:**
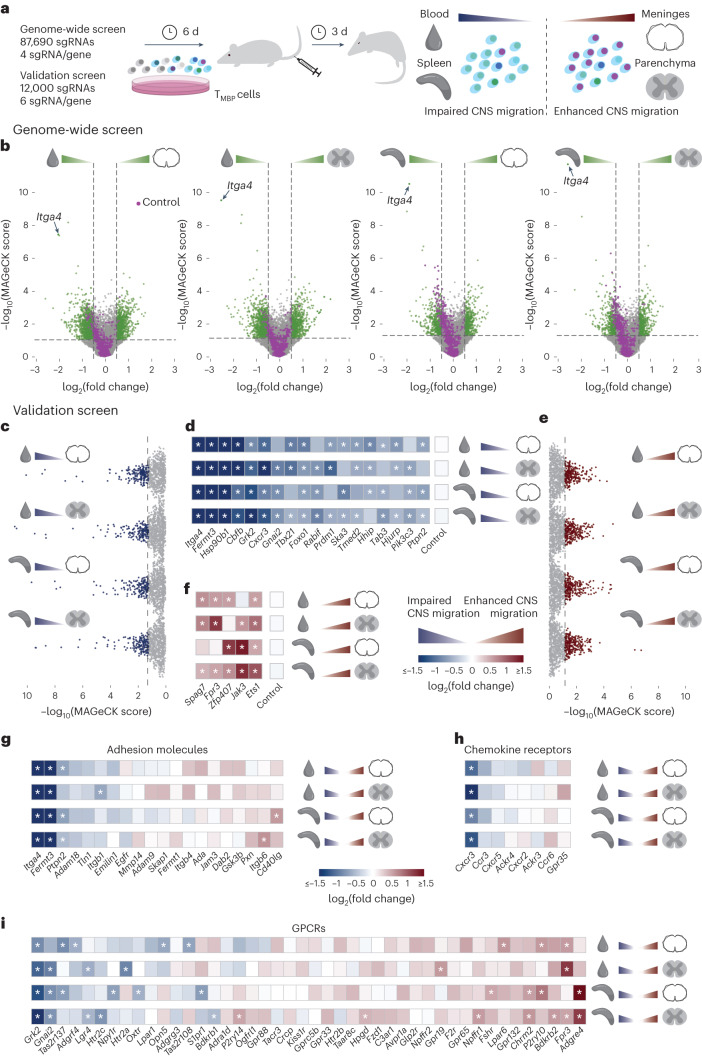
Genome-wide CRISPR screen identifies genes essential for autoreactive T cell migration to the CNS. **a**, Experimental design. T_MBP_ cells were transduced in vitro with the genome-wide or validation CRISPR libraries, positively selected, reactivated and injected intravenously into Lewis rats. After 3 d, at disease onset, they were collected from blood, spleen, spinal cord meninges and parenchyma for analysis. **b**, Volcano plots depicting the genome-wide screen results per gene across four tissue comparisons. Green dots represent genes whose KO showed a sizeable change in the ability of T_MBP_ cells to migrate into the CNS. Lilac dots indicate controls. Lines at *P* value = 0.05 and log_2_(fold change) = ±0.5. **c**–**f**, Validation screen results showing the top-ranking genes whose KO showed impaired (**c**) or enhanced (**e**) migration into the CNS, across comparisons, and log_2_(fold change) heat maps showing the genes essential for ‘facilitating’ T_MBP_ cell entry into the CNS (KO impairs migration, blue) (**d**) or for ‘braking’ CNS migration (KO enhances migration, red) (**f**). Essential candidates were defined as detailed in the Methods. **g**–**i**, log_2_(fold change) heat maps depicting the effects of gene KOs on T_MBP_ cells migration in the validation screen for adhesion-related genes (**g**) (GO terms GO.0050901, GO.0033631 and GO.0005178), chemokine receptors (**h**) (GO.0004950) and GPCRs (**i**) (GO.0004930, GO.0004703, GO.0001664 and guanine nucleotide-binding genes of GO.0001664, excluding genes present in GO.0004950 or GO.0004896). For **g**–**i**, only genes of the GO term with a *P* value < 0.05 (**g** and **h**) or *P* < 0.01 (**i**) and ≥3 ‘neg/pos|goodsgrna’ per the validation screen results are shown. Asterisks indicate, for all heat maps: *P* value < 0.05, absolute log_2_(fold change) > 3 standard deviations of the log_2_(fold changes) of the controls and ≥3 ‘neg/pos|goodsgrna’. Source data

**Extended Data Fig. 1:**
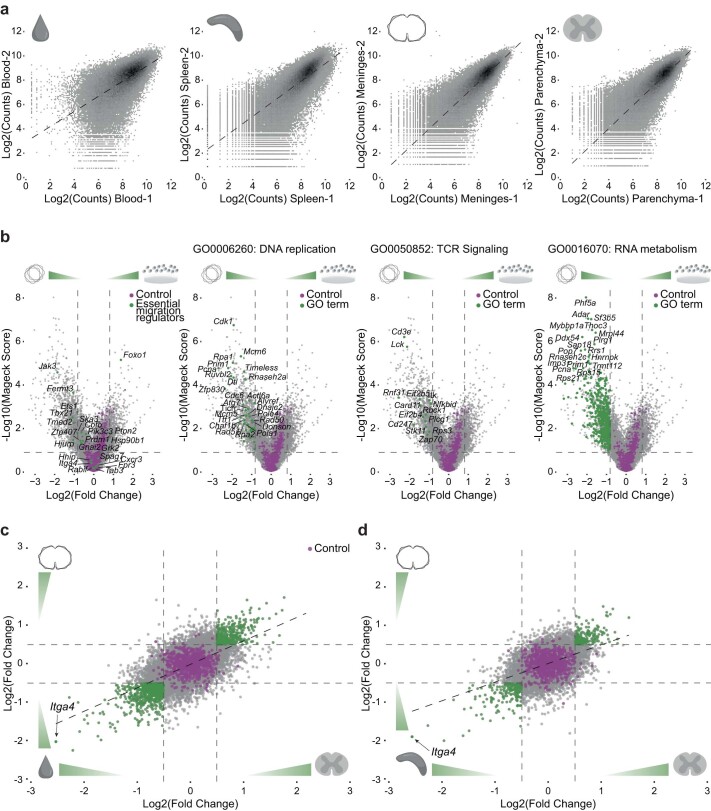
Analysis of the genome-wide CRISPR screen. a, Correlations of the raw sgRNA count values of two representative replicates in (left to right) blood (Pearson correlation index of 0.74), spleen (0.78), meninges (0.86) and parenchyma (0.85). b, Volcano Plots of the genome-wide screen results comparing cultured TMBP cells right before in vivo transfer to the plasmid library, showing in green from left to right the essential regulators of migration as per Fig. 1d,f and genes belonging to the GO terms “DNA replication”, “TCR signaling” and “RNA metabolism”, many of which are depleted in cultured T cells compared to the plasmid library. Lilac, controls. Lines indicate p-value = 0.05 and log2(Fold Change) = ±3 times the standard deviation of the controls. c, Correlation of log2(Fold Change) of genome-wide CRISPR screen for meninges vs. blood compared to parenchyma vs. blood (Pearson correlation index 0.62) and d, meninges vs. spleen compared to parenchyma vs. spleen (0.48). Green, correlating genes whose KO shows a sizeable change in the ability of TMBP cells to migrate into the CNS. Lilac, controls. Source data

To study the molecular regulation of autoreactive T cell trafficking to the CNS in MS, we used a rat EAE model, in which an MS-like disease is induced by the transfer of MBP-reactive T (T_MBP_) cells^4,7,18^. We first conducted a genome-wide screen to identify candidate molecules whose deletion significantly enhanced or impaired T_MBP_ cell migration into the CNS. We transduced T_MBP_ cells in a first step with the Cas9 nuclease and enhanced green fluorescent protein (EGFP), and then with blue fluorescent protein (BFP) and a genome-wide CRISPR library containing 87,690 single guide RNAs (sgRNAs) targeting 21,410 genes and 396 microRNAs (miRNAs), as well as 800 non-targeting (NT) control sgRNAs. We included 300 × 10^6^ T cells per replicate transduced at a multiplicity of infection (MOI) < 0.3 so that statistically most T cells would contain no more than one sgRNA^19^, each different sgRNA would be present in about 1,000 T cells (1,000× coverage) and each gene would be targeted by four different sgRNAs. After 6 d, these cells were injected intravenously into naïve Lewis rats. Three days later, at the time of first EAE symptoms, we isolated T cells from the blood, spleen, spinal cord meninges and parenchyma (Fig. 1a) and used next-generation sequencing (NGS) followed by bioinformatic analysis with the MAGeCK software^20^ to compare the sgRNA distribution for each gene between each of the peripheral and CNS compartments (Fig. 1b). To the list of genes showing a differential distribution between compartments, we applied a set of selection criteria based on effect size and statistical significance (Methods and Supplementary Table 1), leading to the identification of 1,961 candidate target genes for a subsequent validation screen. *Itga4*, which encodes the target of the therapeutic monoclonal antibody natalizumab, α4-integrin, was one of the most-depleted genes in all comparisons of peripheral compartments versus the CNS (Fig. 1b), validating the ability of this approach to identify clinically relevant molecules. Furthermore, we found that many of the same genes appeared to regulate the entry of autoreactive T cells to both the meninges and the spinal cord parenchyma in EAE (Extended Data Fig. 1).

**Extended Data Fig. 2:**
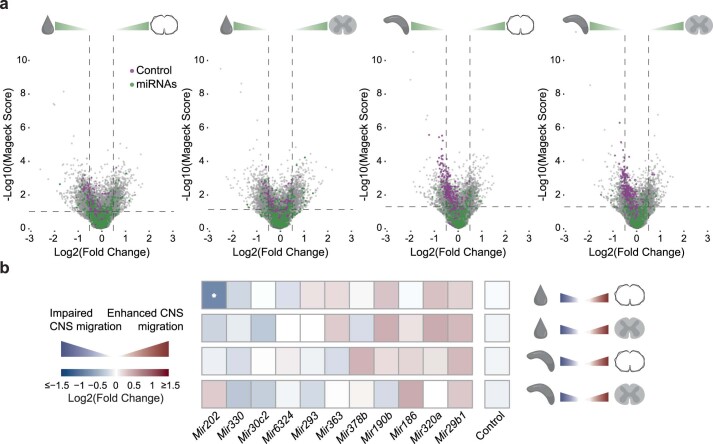
miRNA based regulation of T cell migration to the CNS. a, Genome-wide screen results across different tissue compartments, comparing (from left to right) meninges or parenchyma to blood and meninges or parenchyma to spleen. Lines indicate p-value = 0.05 and log2(Fold Change) = ±0.5. Green, miRNAs, lilac controls. b, Results from the validation screen showing the miRNAs that were included, comparing (from top to bottom) meninges or parenchyma to blood and meninges or parenchyma to spleen. Star indicates p-value < 0.05, absolute log2(Fold Change) > 3 standard deviations of the sample and ≥ 3 “neg/pos|goodsgrna”. Source data

**Extended Data Fig. 3:**
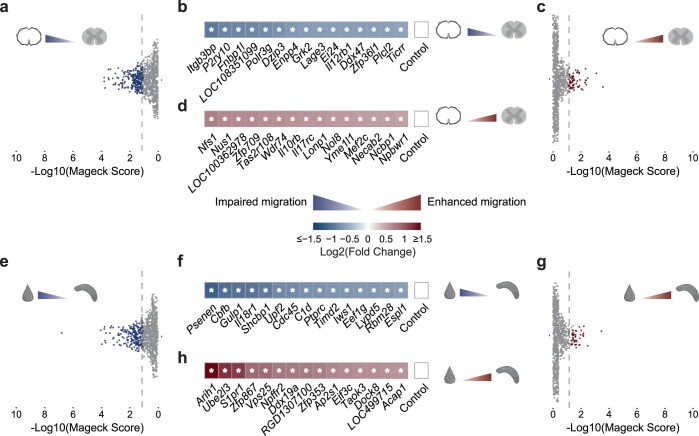
Genes regulating differential migration between spinal cord parenchyma and meninges or between spleen and blood. a-d, Validation screen results showing the top-ranking genes whose KO showed impaired (a) or enhanced (c) migration of TMBP cells to the spinal cord parenchyma compared to the meninges, and b,d, log2(Fold Change) heatmaps showing the genes essential for “facilitating” TMBP cell entry to the parenchyma compared to the meninges (KO impairs migration, blue) (b) or for “braking” migration to the parenchyma compared to meninges (KO enhances migration, red) (d). e-h, Validation screen results showing the top-ranking genes whose KO showed impaired (e) or enhanced (g) migration of TMBP cells to the spleen compared to blood, and f,h, log2(Fold Change) heatmaps showing the genes essential for “facilitating” TMBP cell entry to the spleen from the blood (KO impairs migration, blue) (f) or for “braking” spleen migration (KO enhances migration, red) (h). Stars indicate, for all heatmaps, p-value < 0.05, absolute log2(Fold Change) > 3 standard deviations of the log2(Fold Changes) of the controls and ≥ 3 “neg/pos|goodsgrna”. Source data

In a validation screen, we repeated the adoptive transfer experiment for the 1,961 candidate gene targets identified in the genome-wide screen, but this time targeted more stringently with 6 sgRNAs per gene and with stricter selection criteria at the data analysis stage (for details, see Methods and Supplementary Table 2). Based on the relative fold change of the differential distribution and its robustness across sgRNAs and different compartments, we identified 18 essential ‘facilitators’ that are required for autoreactive T cell migration to the CNS (Fig. 1c,d) and 5 essential ‘brakes’ that limit T cell trafficking to the CNS (Fig. 1e,f). In contrast, none of the miRNAs fulfilled these criteria, indicating that no single miRNA is essential for either promoting or preventing T cell entry to the CNS (Extended Data Fig. 2). Furthermore, our approach also identified genes that differentially regulated the distribution of CD4^+^ T cells within the central (meninges versus spinal cord parenchyma) or peripheral (spleen versus blood) compartments; however, most of the genes involved showed comparably moderate fold changes (Extended Data Fig. 3).

The top-ranked hits of the 18 essential facilitators of CD4^+^ T cell entry to the CNS belonged the Gene Ontology (GO) terms related to ‘adhesion molecules’ (most prominently *Itga4* and the functionally related *Fermt3* gene; Fig. 1g), ‘chemokine receptors’ (in particular *Cxcr3*; Fig. 1h) and ‘G-protein-coupled receptor (GPCR)-related proteins’ (such as *Grk2* and *Gnai2*; Fig. 1i). Notably, some of the essential genes also encoded transcriptional regulators (Fig. 1d) including: *Cbfb*, which has so far been primarily implicated in T cell differentiation^21^; *Tbx21/*T-bet, which controls genes important for the function of the T_H_1 subset of helper T cells^22^; *Foxo1*, encoding a prominent regulator of metabolic T cell fitness^23^; and *Prdm1*, which is critical for both regulatory and cytotoxic T cell properties^24,25^. Only one essential regulator, *Ska3*, which encodes a component of the microtubule-binding SKA1 complex^26^, has a direct link to the cytoskeleton, indicating that most of the genes identified in our screen do not encode proteins that limit T cell mobility in general, but rather selectively impede trafficking from peripheral to CNS compartments.

### The adhesion module: ITGA4, FERMT3 and HSP90B1

**Fig. 2:**
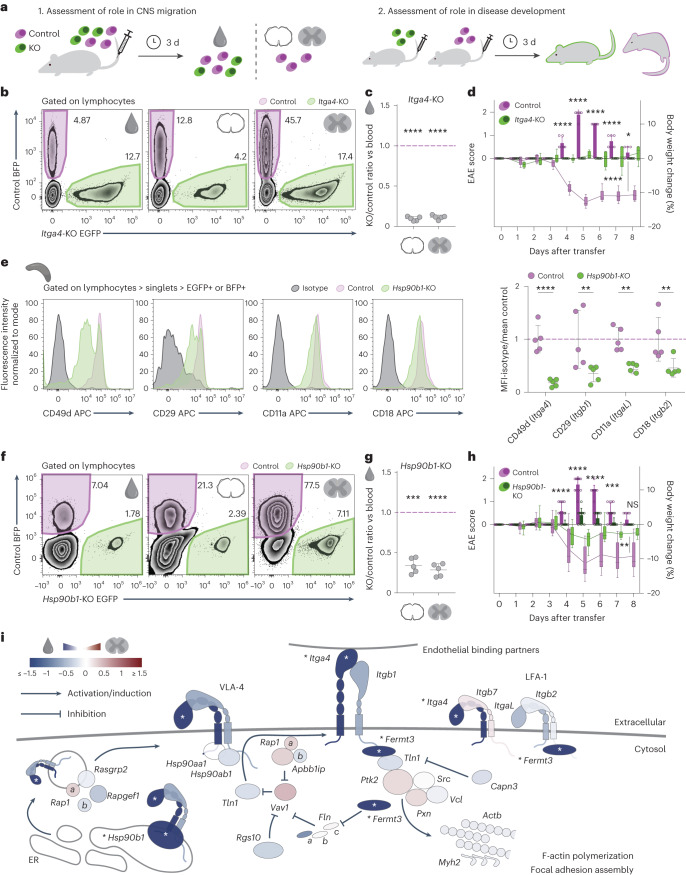
Functional validation of the adhesion module. **a**, Experimental design. KO-EGFP^+^ T_MBP_ cells were (1) co-transferred with control-BFP^+^ T_MBP_ cells or (2) transferred alone. (1) The relative proportions of control and KO cells in blood and CNS tissues were assessed by flow cytometry 3 d after transfer or (2) the animals were kept longer to assess disease development. **b**,**f**, Representative flow cytometry plots of T_MBP_ cells from blood, meninges and parenchyma after co-transfer with control and *Itga4*-KO (**b**) or *Hsp90b1*-KO (**f**) cells. **c**,**g**, Migratory phenotype of *Itga4*-KO (**c**) or *Hsp90b1*-KO (**g**) cells compared to control, shown as the ratio of KO to control T_MBP_ cells in meninges (left) or parenchyma (right) normalized to the KO/control ratio in blood. *n* = 5 rats. **d**,**h**, EAE score (bars) and weight changes (line) of control and *Itga4*-KO (**d**) or *Hsp90b1*-KO (**h**) injected animals; *n* = 6 rats per group for **d** and *n* = 12 control, *n* = 11 KO rats for **h**. **e**, Representative flow cytometry plots of integrin surface stainings of *Hsp90b1*-KO and control T_MBP_ cells isolated from the spleens of co-transferred rats 3 d after transfer; gray indicates isotype control, lilac indicates control, and green indicates KO. Right, quantification of the median fluorescence intensity (MFI) with isotype background subtraction normalized to the mean control intensity. *n* = 5 rats. **i**, Schematic of the adhesion module with genes color coded based on the parenchyma versus blood comparison. Asterisks indicate significance across at least three pairwise tissue comparisons (Methods). **c**,**g**, One-sample *t*-test against hypothetical mean = 1; **d**,**h**, bars and dots, EAE score; box-and-whiskers plot and line, body weight change; repeated-measures two-way analysis of variance (ANOVA; days 3–8 for disease score; **d**, *F* = 1094, *P* < 0.0001; **h**, *F* = 154.6, *P* < 0.0001, and days 0–8 for weight changes; **d**, *F* = 100.3, *P* < 0.0001; **h**, *F* = 11.89, *P* = 0.0024) and Sidak’s multiple-comparison test only for EAE score; **e**, two-way ANOVA (*F* = 51.13, *P* < 0.0001) with multiple comparisons with the two-stage linear step-up procedure of Benjamini, Krieger and Yekutieli. Figures show the mean ± s.d. Not significant (NS), *P* > 0.05, *P < 0.05, **P < 0.01, ***P < 0.001, ****P < 0.0001. Source data

To further characterize the ‘adhesion module’, we first validated the effects of *Itga4*-knockout (KO) on T cell migration by CRISPR editing. We co-transferred *Itga4*-KO T_MBP_ cells expressing EGFP with T_MBP_ cells edited with a NT control sgRNA and expressing BFP (control T_MBP_ cells) into rats and collected cells from the blood and spinal cord meninges and parenchyma 3 d later (Fig. 2a). By flow cytometry, we confirmed that *Itga4* deletion significantly reduced T_MBP_ cell migration into the rodent CNS (Fig. 2b,c). Accordingly, the transfer of *Itga4*-KO T_MBP_ cells alone failed to induce disease symptoms in the recipient rats, while rats that received control T_MBP_ cells showed the expected disease course (Fig. 2a,d).

**Extended Data Fig. 4:**
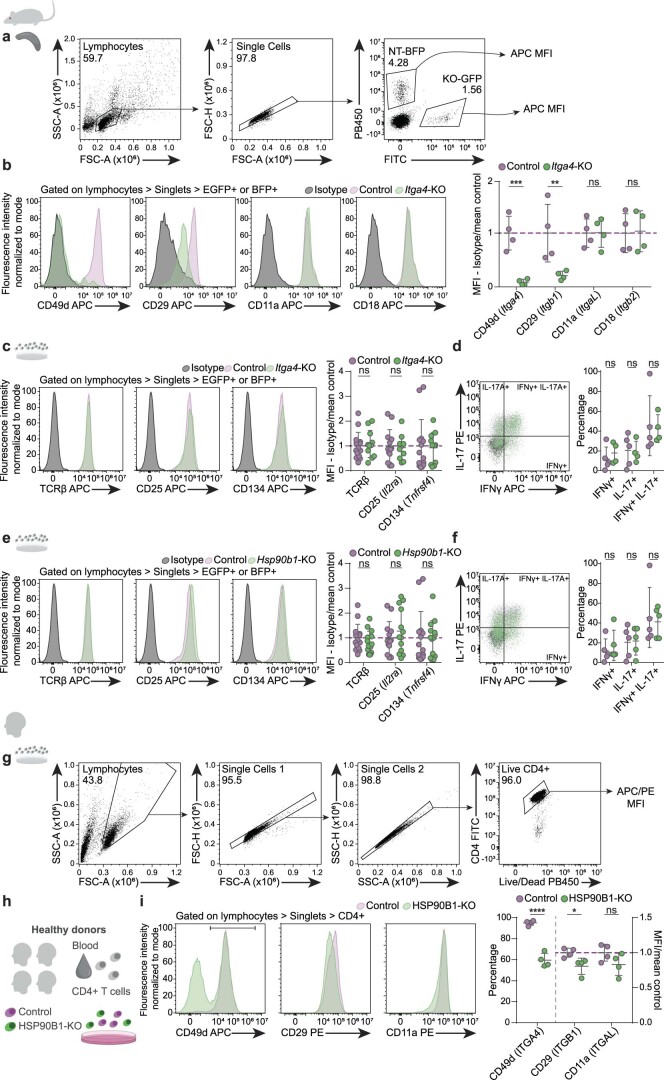
Effects of editing components of the adhesion module in rat TMBP cells and human CD4 + T cells. a, Gating strategy for rat T cell experiments. An example spleen sample is shown. b, Representative flow cytometry plots of integrin surface stainings of Itga4 -KO and control TMBP cells isolated from the spleen of co-transferred rats 3 days post-transfer, grey isotype control, lilac control, green KO. Right, quantification of the MFI with isotype background subtraction normalized to the mean control intensity. n = 4 rats. c,e, Representative flow cytometry plots of activation marker surface stainings from TMBP cells (control and Itga4-KO (c) or Hsp90b1-KO (e)) in vitro at 2 days post restimulation; grey, isotype control; lilac, control; green, KO. Right, quantification of the MFI with isotype background subtraction normalized to the mean control intensity. n = 14 independent experiments for control and Hsp90b1-KO, TCRβ and CD134; n = 13 control and Hsp90b1-KO, CD25; n = 11 Itga4-KO, TCRβ and CD134; n = 10 Itga4-KO, CD25. d,f, Representative flow cytometry plots of intracellular cytokine staining from TMBP cells (control and Itga4-KO (d) or Hsp90b1-KO (f)) in vitro at 1 day post restimulation, grey isotype control, lilac control, green KO. Right, quantification of the percentages. n = 5 independent experiments for control and Hsp90b1-KO; n = 4 for Itga4-KO. g, Gating strategy for human T cell experiments. h, Experimental design of human HSP90B1-KO CD4 + T cell surface stainings. i, Representative flow cytometry plots of integrin surface stainings from in vitro HSP90B1-KO human CD4 + T cells; lilac, control, green, KO. Right, quantification of the percentage or of the MFI normalized to the mean control intensity. n = 4 donors. b-f,i MFI, two-way ANOVA (b, F = 13.37, P = 0.0013; c, F = 0.01785, P ns; d, F = 0.00591, P ns; e, F = 0.0119, P ns; f, F = 0.01325, P ns; i, F = 9.179, P = 0.0105) with multiple comparisons with the two-stage linear step-up procedure of Benjamini, Krieger and Yekuteli, i, percentage, unpaired t-test. Control FACS datapoints for in vitro surface (c,e) and intracellular stainings (d,f) are the same for all KOs, which were analysed in parallel (cf. Extended Data Figs. 5, 6 and 7). Figures show mean ± s.d, P > 0.05 ns (non-significant), P < 0.05 *, P < 0.01 **, P < 0.001 ***, P < 0.0001 ****. Source data

Considering other adhesion-related genes that might work alongside *Itga4* to drive T cell migration into the CNS, we next assessed the effects of knocking-out *Hsp90b1*. The HSP90 heat shock protein family has been proposed to act as chaperones and allow correct folding of integrins^27^. Here we now show that one member, HSP90B1, in particular is essential for α4-integrin actions as *Hsp90b1*-KO T_MBP_ cells displayed a marked reduction of α4-integrin surface expression as well as a moderate reduction of the surface expression of ß1-integrin and the lymphocyte function associated-antigen 1 (LFA-1) components αL-integrin and ß2-integrin (Fig. 2e) compared to control T_MBP_ cells. The moderate effects of HSP90B1 on ß1-integrin expression might be secondary to the reduced surface presence of α4-integrin as the deletion of *Itga4* also reduced the expression of its binding partner ß1-integrin, while it did not result in marked changes of αL-integrin and ß2-integrin surface expression (Extended Data Fig. 4). Notably, this chaperone function of HSP90B1 appears to be conserved in humans, as CRISPR editing of HSP90B1 in human CD4^+^ T cells isolated from peripheral blood resulted in a marked reduction of the surface expression of CD49d/α4-integrin (Extended Data Fig. 4). In line with the marked effects of HSP90B1 on α4-integrin surface expression, we observed a moderate yet significant reduction in T cell trafficking to spinal cord meninges and parenchyma (Fig. 2f,g), and a significantly milder EAE course (Fig. 2h) when *Hsp90b1*-KO T_MBP_ cells were transferred into rats. Like *Itga4*-KO T_MBP_ cells, *Hsp90b1*-KO T_MBP_ cells did not show altered expression of activation markers or the cytokines interferon (IFN)-γ and interleukin (IL)-17A (Extended Data Fig. 4).

Taken together, these results outline the functional T cell adhesion module that contains α4-integrin, its intracellular binding partner kindlin3 (encoded by *Fermt3*, identified in our validation screen and with an established role in the intracellular activation of integrins^28^), and its chaperone HSP90B1 (Fig. 2i). Notably, all other adhesion-related genes in our screen are nonessential (at least by our strict definition) and even deletion of *Itgb1* that encodes ß1-integrin, which pairs with α4-integrin to form VLA-4, the binding partner for endothelial VCAM-1, shows only a mild reduction in T cell migration to the CNS, likely because it can be replaced by other ß-integrins such as ß7-integrin^29^.

### The chemotaxis module: CXCR3, GNAI2 and TBX21

**Fig. 3:**
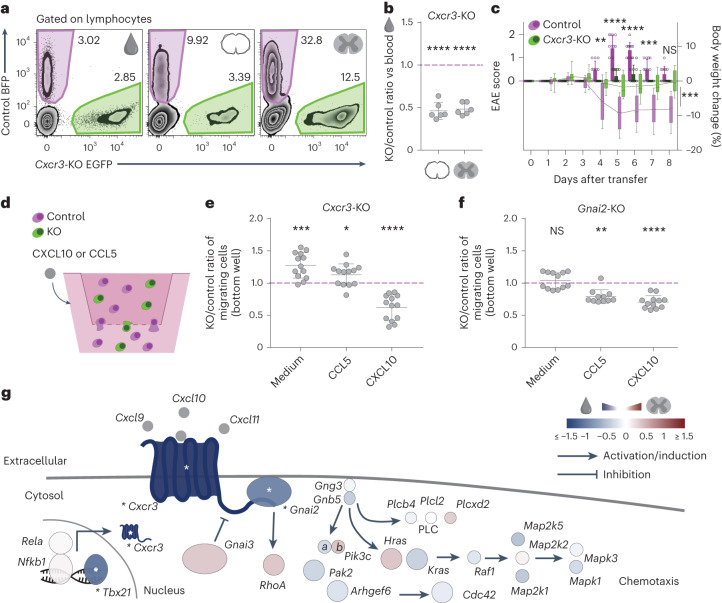
Functional validation of the chemotaxis module. **a**, Representative flow cytometry plots of T cells from blood, meninges and parenchyma after a co-transfer experiment with control and *Cxcr3*-KO cells. **b**, Migratory phenotype of *Cxcr3*-KO cells compared to control, shown as the ratio of KO and control T_MBP_ cells in meninges (left) or parenchyma (right) normalized to the KO/control ratio in blood. *n* = 6 rats. **c**, EAE score (bars) and weight changes (line) of control and *Cxcr3*-KO injected animals; *n* = 12 rats per group. **d**, Experimental design of in vitro transwell T_MBP_ cell migration experiments. Cells were seeded in the top chamber with medium, and control medium or chemokines were added to the bottom chamber. **e**,**f**, In vitro migration in response to chemokine gradient of *Cxcr3*-KO (**e**) cells and *Gnai2*-KO (**f**) cells normalized to control. *n* = 13 transwell assays for *Cxcr3*-KO; *n* = 12 for *Gnai2*-KO medium and CXCL10; *n* = 11 for *Gnai2*-KO CCL5. **g**, Schematic of the CNS chemotaxis module centered around CXCR3. Genes are color coded based on the CRISPR screen phenotype in the parenchyma versus blood comparison. Asterisks indicate significance across at least three pairwise tissue comparisons (Methods). **b**,**e**,**f**, One-sample *t*-test or Wilcoxon signed-rank test against hypothetical mean = 1; **c**, bars and dots, EAE score; box-and-whiskers plot and line, body weight change; repeated-measures two-way ANOVA (days 3–8 for disease score (*F* = 111.5, *P* < 0.0001) and days 0–8 for weight changes (*F* = 18.22, *P* = 0.0003)) and Sidak’s multiple-comparison test only for EAE score. Figures show the mean ± s.d. NS, *P* > 0.05, *P < 0.05, **P < 0.01, ***P < 0.001, ****P < 0.0001. Source data

**Extended Data Fig. 5:**
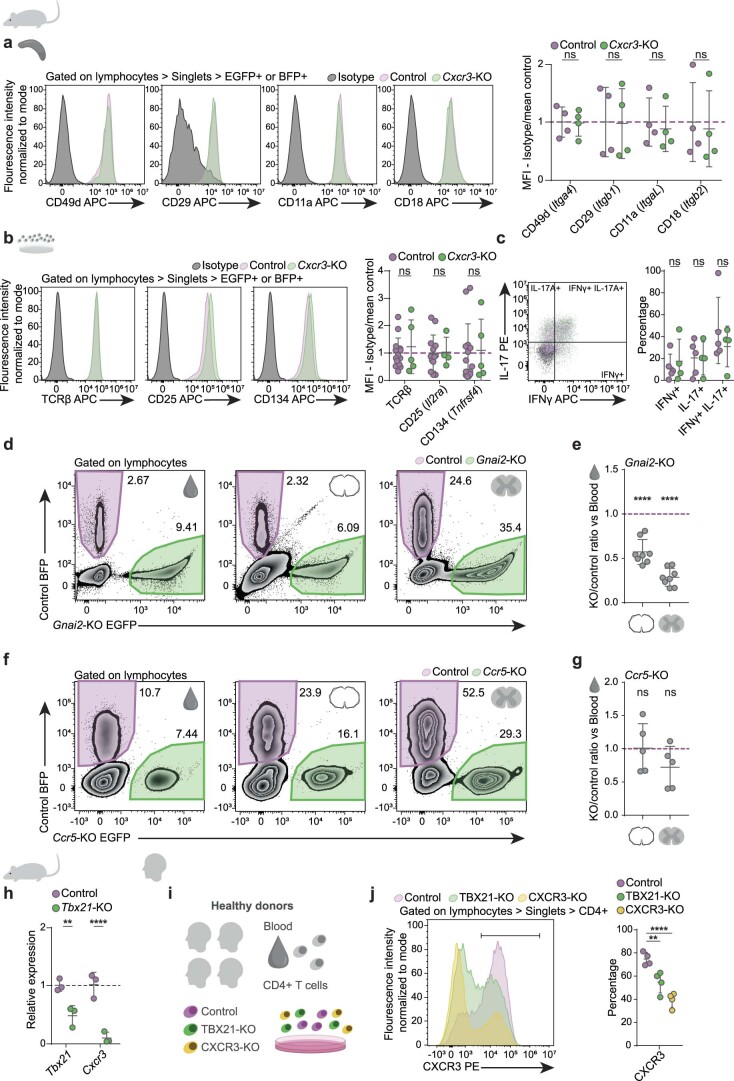
Effects of editing components of the chemotaxis module in rat TMBP cells and human CD4 + T cells. a, Representative flow cytometry plots of integrin surface stainings of Cxcr3-KO and control TMBP cells isolated from the spleen of co-transferred rats 3 days post-transfer; grey, isotype control; lilac, control; green, KO. Right, quantification of the MFI with isotype background subtraction normalized to the mean control intensity. n = 4 rats. b, Representative flow cytometry plots of activation marker surface stainings from TMBP cells (control and Cxcr3-KO) in vitro at 2 days post restimulation; grey, isotype control; lilac, control; green, KO. Right, quantification of the MFI with isotype background subtraction normalized to the mean control intensity. n = 14 independent experiments for control TCRβ and CD134; n = 13 control for CD25; n = 5 for Cxcr3-KO, TCRβ and CD134; n = 4 for Cxcr3-KO, CD25. c, Representative flow cytometry plots of intracellular cytokine staining from TMBP cells (control and Cxcr3-KO) in vitro at 1 day post restimulation; grey, isotype control; lilac, control; green, KO. Right, quantification of the percentages. n = 5 independent experiments for control; n = 4 for Cxcr3-KO. d,f, Representative flow cytometry plots of T cells from blood, meninges and parenchyma after a co-transfer experiment with control and Gnai2-KO (d) or Ccr5-KO (f) TMBP cells. e,g, Migratory phenotype of Gnai2-KO (e) or Ccr5-KO (g) cells compared to control, shown as the ratio of KO /control in meninges (left) or parenchyma (right) normalized to the KO/control ratio in blood. n = 8 rats for Gnai2-KO, n = 5 for Ccr5-KO. h, qPCR results in Tbx21-KO and control cells in vitro. n = 3 samples per group. i, Experimental design of human TBX21-KO and CXCR3-KO control T cell surface stainings. j, Representative flow cytometry plots of control and KO cells stained for CXCR3. Right, quantification of the percentage. n = 4 donors. a,b,c, ordinary two-way ANOVA (a, F = 0.1481, P ns; b, F = 0.2169, P ns; c, F = 0.172, P ns) with multiple comparisons with the two-stage linear step-up procedure of Benjamini, Krieger and Yekuteli. e,g, one sample t-test against hypothetical mean = 1. h, multiple paired t-tests with two-stage linear step-up procedure of Benjamin, Krieger and Yekutieli multiple testing correction. j, ordinary one-way ANOVA (F = 23.96, P = 0.0002) with multiple comparisons with the two-stage linear step-up procedure of Benjamini, Krieger and Yekuteli. Control FACS datapoints for in vitro surface (b) and intracellular stainings (c) are the same for all KOs, which were analysed in parallel (cf. Extended Data Figs. 4, 6 and 7). Figures show mean ± s.d, P > 0.05 ns (non-significant), P < 0.05 *, P < 0.01 **, P < 0.001 ***, P < 0.0001 ****. Source data

CXCR3, the receptor for CXCL9, CXCL10 and CXCL11, was the only top hit in our screen among the chemokine receptor family, despite previous work implicating both CXCR3 and CCR5 as regulators of T cell trafficking in the leptomeninges^7^. We first confirmed the essential role of CXCR3, showing that *Cxcr3*-KO T_MBP_ cells but not *Ccr5*-KO T_MBP_ cells migrated significantly less to the spinal cord meninges and parenchyma compared to co-transferred control T_MBP_ cells (Fig. 3a,b and Extended Data Fig. 5). Accordingly, transfer of *Cxcr3*-KO T_MBP_ cells induced significantly milder disease than did control T_MBP_ cells (Fig. 3c). This effect is likely related to the altered migratory capacities of these cells as *Cxcr3*-KO T_MBP_ cells did not show significant changes in the expression of adhesion molecules, activation markers or the cytokines IFN-γ and IL-17A (Extended Data Fig. 5).

Further analysis of this ‘chemotaxis module’ suggests that, in addition to CXCR3, two key proteins might be involved in chemoattraction of T cells into the CNS: the guanine nucleotide-binding protein GNAI2, which is linked to the intracellular transduction of signals induced by the CXCL9, CXCL10 and CXCL11 chemokines^30^ and TBX21/T-BET, which controls the expression of the CXCR3 receptor^22^. To assess the role of GNAI2 in CXCR3-mediated migration, we compared the migration of *Cxcr3*-KO and *Gnai2*-KO T_MBP_ cells to control T_MBP_ cells in a transwell chemotaxis assay. We found that *Cxcr3*-KO T cells showed altered transmigration in response to the CXCR3 ligand CXCL10, but not to the unrelated chemokine CCL5, while *Gnai2*-KO reduced migration toward both chemokines (Fig. 3d–f). This indicates that GNAI2 transduces the effects of CXCR3 on T cell migration and indeed we observed that *Gnai2*-KO T_MBP_ cells showed impaired migration to meninges and spinal cord parenchyma after transfer (Extended Data Fig. 5). Furthermore, CRISPR editing of the transcriptional regulator TBX21/T-BET in rat T_MBP_ cells and human CD4^+^ T cells confirmed its critical role for controlling CXCR3 expression, which is conserved across species (Extended Data Fig. 5). Thus, based on these analyses, the chemotaxis module comprises CXCR3, GNAI2 and the transcription factor TBX21/T-BET (Fig. 3g).

### The egress module: GRK2 controls T cell trafficking via S1PR1

**Fig. 4:**
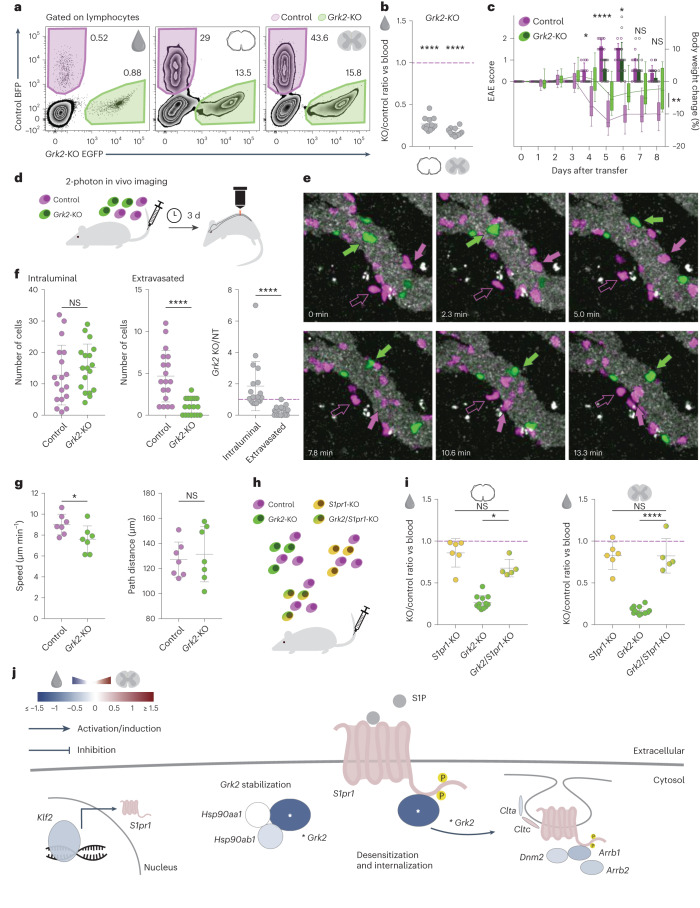
Functional validation of the egress module. **a**, Representative flow cytometry plots of T cells from blood, meninges and parenchyma after a co-transfer experiment. **b**, Migratory phenotype of *Grk2*-KO cells compared to control, shown as the ratio of KO/control in meninges (left) or parenchyma (right) normalized to the KO/control ratio in blood. *n* = 12 rats. **c**, EAE score (bars) and weight changes (line) of control and *Grk2*-KO injected animals; *n* = 15 rats per group. **d**, Experimental design for intravital two-photon imaging. **e**, Time-lapse images tracking cells along the meningeal vasculature. Lilac indicates control cells, and green indicates *Grk2*-KO cells; filled arrows indicate cells inside the blood vessel lumen, and empty arrows indicate extravasated cells. **f**, Distribution of cells in the leptomeninges vasculature. Left, intraluminal cell count; middle, extravasated cell count; right, ratio of KO/control cells derived from data in the previous two plots. *n* = 3 animals, 5–7 images per animal, for a total *n* = 18 images. **g**, Analysis of speed (left) and path distance (right) of cells crawling in the blood vessels. Data summarized per movie, *n* = 3 animals, 2–3 movies per animal, 15–76 cells per condition per movie, for a total *n* = 7 movies. **h**, Experimental design. **i**, Migratory phenotype of KO T_MBP_ cells compared to control T_MBP_ cells, shown as the ratio of KO to control in meninges (left) or parenchyma (right) divided by the KO/control ratio in blood. *n* = 6 rats for *S1pr1*-KO; *n* = 12 rats for *Grk2*-KO same data as in **b**; *n* = 5 rats for *Grk2*/*S1pr1*-KO. **j**, Schematic of the egress module with genes color coded based on the parenchyma versus blood comparison. Asterisks indicate significance across at least three pairwise tissue comparisons (Methods). **b**, One-sample *t*-test against hypothetical mean = 1; **c**, bars and dots, EAE score; box-and-whiskers plot and line, body weight change; repeated-measures two-way ANOVA (days 3–8 for disease score (*F* = 13.82, *P* = 0.0009) and days 0–8 for weight changes (*F* = 12.69, *P* = 0.0013)) and Sidak’s multiple-comparison test only for EAE score; **f**,**g**, Paired parametric *t*-test or Wilcoxon matched-pairs signed-rank test; **i**, Kruskal–Wallis test (Kruskal–Wallis = 17.01, *P* = 0.0002) with Dunn’s multiple-comparison test for meninges, and one-way ANOVA (*F* = 75.6, *P* < 0.0001) with Turkey’s multiple-comparison test for parenchyma. Figures show the mean ± s.d. NS, *P* > 0.05, *P < 0.05, **P < 0.01, ***P < 0.001, ****P < 0.0001. Source data

**Extended Data Fig. 6:**
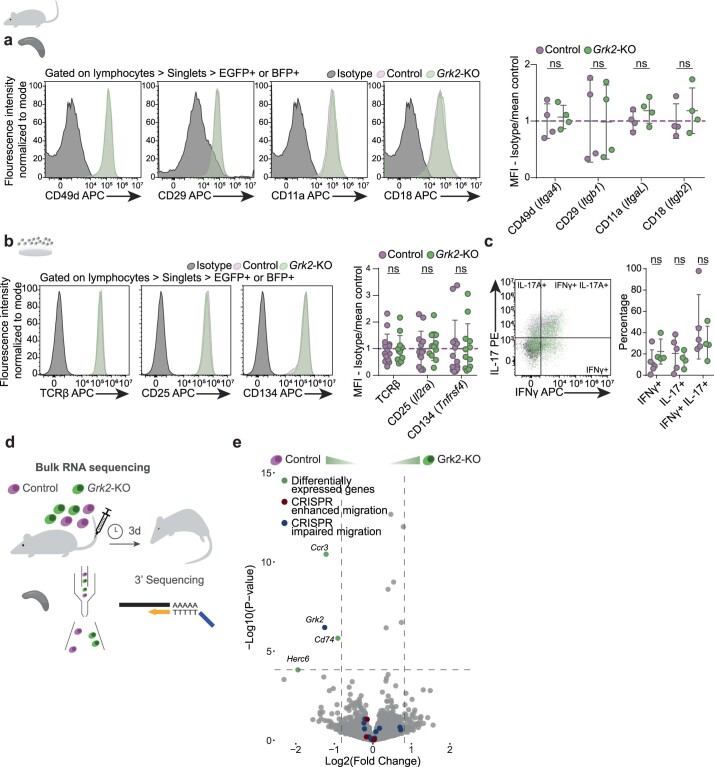
Effects of editing Grk2 on adhesion, activation, cytokine production and transcriptional profile of rat TMBP cells. a, Representative flow cytometry plots of integrin surface stainings of Grk2-KO and control TMBP cells isolated from the spleen of co-transferred rats 3 days post-transfer; grey, isotype control; lilac, control; green, KO. Right, quantification of the MFI with isotype background subtraction normalized to the mean control intensity. n = 4 rats. b, Representative flow cytometry plots of activation marker surface stainings from TMBP cells (control and Grk2-KO) in vitro at 2 days post restimulation; grey, isotype control; lilac, control; green, KO. Right, quantification of the MFI with isotype background subtraction normalized to the mean control intensity. n = 14 independent experiments for control TCRβ and CD134; n = 13 for control, CD25; n = 12 for Grk2-KO, TCRβ and CD134; n = 11 for Grk2-KO, CD25. c, Representative flow cytometry plots of intracellular cytokine staining from TMBP cells (control and Grk2-KO) in vitro at 1 day post restimulation; grey, isotype control; lilac, control; green, KO. Right, quantification of the percentages. n = 5 independent experiments for control; n = 4 for Grk2-KO. d, Experimental design of the bulk RNAseq experiment of Grk2-KO and control TMBP cells from the spleen of co-transferred animals. e, Volcano plot of the RNAseq results of Grk2-KO cells compared to control TMBP cells, lines indicate adjusted p-value = 0.05 and log2(Fold Change) = ±3 standard deviations of the sample. Green, significantly differentially expressed genes; blue, essential genes “facilitating” CNS migration in TMBP cells (“CRISPR impaired migration”); red, essential genes “braking” CNS migration (“CRISPR enhanced migration”) from Fig. 1d,f. Control FACS datapoints for in vitro surface (b) and intracellular stainings (c) are the same for all KOs, which were analysed in parallel (cf. Extended Data Figs. 4, 5 and 7). a,b,c, two-way ANOVA (a, F = 0.5042, P ns; b, F = 0.06375, P ns; c, F = 0.2459, P ns) with multiple comparisons with the two-stage linear step-up procedure of Benjamini, Krieger and Yekuteli. Figures show mean ± s.d, P > 0.05 ns (non-significant), P < 0.05 *, P < 0.01 **, P < 0.001 ***, P < 0.0001 ****. Source data

Among the GPCR-related proteins, the GPCR kinase 2 (GRK2) is the dominant hit in our screen with one of strongest effect sizes of any regulator outside the adhesion module (Fig. 1d,i). This was unexpected as GRK2 haploinsufficiency in mice had been previously related to an earlier disease onset in the EAE model^31^. Here we now established the essential role of GRK2 for CNS migration by transferring CRISPR-edited *Grk2*-KO T_MBP_ cells, which showed a significant reduction in their capacity to reach either spinal cord meninges or parenchyma (Fig. 4a,b), and induced significantly milder disease symptoms (Fig. 4c), compared to control T_MBP_ cells. As GRK2 has multiple potential target substrates and mechanisms^32^, we next asked which aspect of the transmigration process was affected by loss of GRK2. We performed in vivo multiphoton imaging of the rat spine after co-injection of *Grk2*-KO T_MBP_ cells expressing EGFP and control T_MBP_ cells expressing BFP (Fig. 4d). By tracking the location and movement of individual GRK2-deficient and GRK2-competent autoreactive T cells in the meninges, we found that GRK2 loss primarily affects the distribution of T cells between the intravascular and extravascular CNS compartments (Fig. 4e,f and Supplementary Movies 1 and 2), while the speed and the path lengths of crawling T cells along the vascular surface were mostly unaffected (Fig. 4g). Together with our observation that *Grk2*-KO T_MBP_ cells showed neither alterations in the expression of adhesion molecules, activation markers or the cytokines IFN-γ and IL-17A nor marked changes of their transcriptomic profile (Extended Data Fig. 6), this argues that loss of GRK2 alters the responsiveness of T cells to signals that determine their diapedesis but does not affect either their adhesion to the endothelial cells or their overall activation status or movement properties. These results are reminiscent of the altered trafficking of GRK2-deficient B cells between blood and lymph nodes that has been related to the desensitization of the S1PR1 receptors by GRK2 (ref. ^33^). Therefore, we next asked whether this mechanism was also important for T cell migration to the CNS using co-transfer experiments (Fig. 4h). We found that, while *Grk2*-KO T cells showed a marked impairment in trafficking from blood to either CNS compartment, these deficits were ameliorated if T cells lacked both *Grk2* and *S1pr1* (Fig. 4i). As trafficking from the blood to CNS was unaltered in T_MBP_ cells deficient for *S1pr1* alone (Fig. 4i), these findings demonstrate that the essential contribution of GRK2 to the CNS entry of T cells is mediated via S1PR1 (Fig. 4j).

**Fig. 5:**
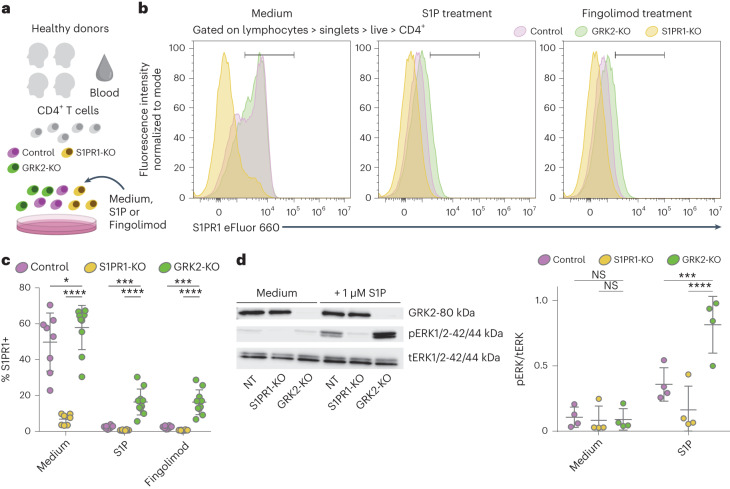
GRK2 mediates S1PR1 internalization in human T cells. **a**, Scheme of the experimental design. CD4^+^ T cells were collected from the buffy coats of healthy donors and CRISPR KOs were generated in vitro. Cells were treated with control medium, S1P or fingolimod, and S1PR1 internalization was measured by fluorescence-activated cell sorting (FACS) surface staining. **b**, Representative flow cytometry plots of control and KO T cells for medium, S1P and fingolimod treatment conditions. **c**, Quantification of the percentage of S1PR1 surface expression after treatment. *n* = 1–3 independent internalization assays from four donors for all groups, for a total of *n* = 8 control and S1PR1-KO, *n* = 10 GRK2-KO S1PR1 internalization assays. **d**, Representative western blot image (left) and quantification (right) of MAPK (ERK1/ERK2) phosphorylation following S1PR1 stimulation with the endogenous ligand S1P. Phospho-ERK1/ERK2 values are shown normalized to total ERK1/ERK2. *n* = 4 donors. Phospho-ERK1/ERK2 and total ERK1/ERK2 blots were run in parallel, GRK2 was stained in the phospho-ERK1/ERK2 blot. **c**,**d**, Two-way ANOVA (**c**, *F* = 83.26, *P* < 0.0001; **d**, *F* = 11.13, *P* = 0.0007) with multiple comparisons with the two-stage linear step-up procedure of Benjamini, Krieger and Yekutieli. Figures show the mean ± s.d. NS, *P* > 0.05, *P < 0.05, **P < 0.01, ***P < 0.001, ****P < 0.0001. Source data

To assess whether this S1PR1–GRK2 axis is similarly operative in human T cells, we used CRISPR-edited human CD4^+^ T cells isolated from the peripheral blood of healthy donors (Fig. 5a). Our results demonstrate that human T cells lacking GRK2 showed partially impaired internalization of S1PR1 in response to both S1P and fingolimod exposure (Fig. 5b,c). Furthermore, when we assessed the effects of GRK2 on the downstream signaling of S1PR1, we observed that the absence of GRK2 resulted in an excessive phosphorylation of ERK1/ERK2 (ref. ^34^) in response to S1P stimulation (Fig. 5d).

Taken together, these data delineate a third functional module that controls the egress of T cells by shifting the balance of attraction between blood and the CNS. This module is centered around the GRK2-mediated phosphorylation of S1PR1, which is likely induced by prolonged exposure of T cells to the S1P ligand in the blood, resulting in desensitization and subsequent internalization of S1PR1 (Fig. 4j). Our results indicate that *Grk2*-KO T cells are unable to egress from the blood even though they still receive attractive signals from the CNS, can adhere to the endothelium and can move inside blood vessels. Remarkably, the actions of S1PR1 agonists, effective therapeutic interventions in MS, also result in impaired S1PR1 signaling^35^. In individuals treated with these agonists, however, it is assumed that the failure of T cells to respond to S1P-mediated attraction to blood sequesters these cells in peripheral lymphoid tissues before they can even reach the CNS. Here too, *S1pr1* KO did not affect the distribution of T_MBP_ cells between blood and CNS compartments (Fig. 4i) but led to an accumulation of these cells in the spleen (*S1pr1*-KO/control ratio in spleen versus blood of 1.37 ± 0.25 (s.d.), *P* = 0.0291, *n* = 5 rats; Extended Data Fig. 3) and parathymic lymph nodes (*S1pr1*-KO/control ratio in lymph nodes versus blood of 5.60 ± 3.24 (s.d.), *P* = 0.0336, *n* = 5 rats). Thus, our findings reveal the delicate regulatory balance that governs S1PR1 signaling during T cell trafficking, with either sustained blocking of signal transmission (as achieved by S1PR1 agonists) or a failure to curb signal transmission (as induced by GRK2 deficiency) preventing encephalitogenic T cells from entering the CNS.

### ETS1 controls responsiveness of autoreactive T cells

**Fig. 6:**
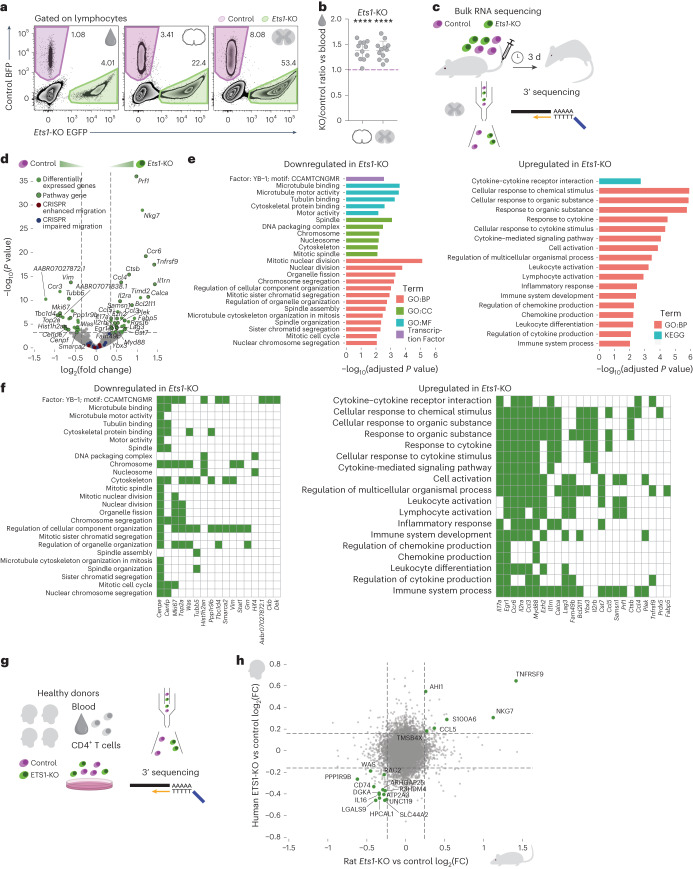
ETS1 inhibits CD4^+^ T cell migration to the CNS. **a**, Representative flow cytometry plots of T cells from blood, meninges and parenchyma after a co-transfer experiment with control and *Ets1*-KO T_MBP_ cells. **b**, Migratory phenotype of *Ets1*-KO cells compared to control, shown as the ratio of KO/control in meninges (left) or parenchyma (right) normalized to the KO/control ratio in blood. *n* = 12 rats. **c**, Experimental design of the bulk RNA sequencing (RNA-seq) of *Ets1*-KO and control cells from the parenchyma of co-transferred animals. **d**, Volcano plot of the RNA-seq results of *Ets1*-KO cells compared to control T_MBP_ cells. Lines at *P* value = 0.05 and log_2_(fold change) = ±3 standard deviations of the log_2_(fold change) of the sample. Green indicates significantly differentially expressed genes, with black circles around those belonging to pathways in **e**; blue indicates essential genes ‘facilitating’ CNS migration in T_MBP_ cells (‘CRISPR impaired migration’); red indicates essential genes ‘braking’ CNS migration (‘CRISPR enhanced migration’) in T_MBP_ cells derived from Fig. 1d,f. **e**, Pathway analysis of the top downregulated and top upregulated pathways in the *Ets1*-KO compared to control T_MBP_ cells. **f**, Significant (adjusted *P* < 0.05 and absolute log_2_(fold change) > 3 standard deviations of the sample) genes belonging to the pathways in **e**. **g**, Experimental design of the bulk RNA-seq of ETS1-KO and control human CD4^+^ T cells. **h**, Correlation between *Ets1*-KO versus control log_2_(fold change) in rat and ETS1-KO versus control log_2_(fold change) in human. Green indicates genes regulated in both species (*P* value < 0.05 in at least one species and absolute log_2_(fold change) > 2 times the standard deviation of the sample in both). **b**, One-sample *t*-test against hypothetical mean = 1. Figures show the mean ± s.d. NS, *P* > 0.05, *P < 0.05, **P < 0.01, ***P < 0.001, ****P < 0.0001. Source data

**Extended Data Fig. 7:**
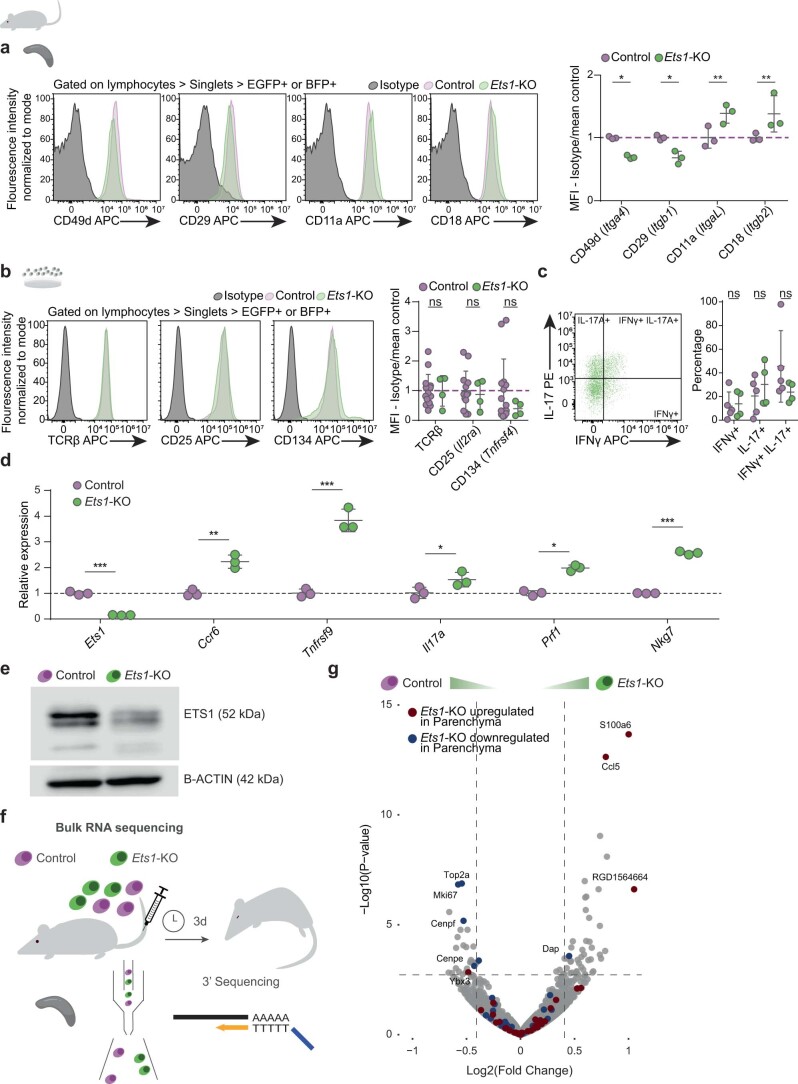
Effects of editing Ets1 on adhesion, activation, cytokine production and transcriptional profile of rat TMBP cells. a, Representative flow cytometry plots of integrin surface stainings of Ets1-KO and control TMBP cells isolated from the spleen of co-transferred rats 3 days post-transfer; grey, isotype control; lilac, control; green, KO. Right, quantification of the MFI with isotype background subtraction normalized to the mean control intensity. n = 3 rats. b, Representative flow cytometry plots of activation marker surface stainings from TMBP cells (control and Ets1-KO) in vitro at 2 days post restimulation; grey, isotype control; lilac, control; green, KO. Right, quantification of the MFI with isotype background subtraction normalized to the mean control intensity. n = 14 independent experiments for control, TCRβ and CD134; n = 13 for control, CD25; n = 4 for Ets1-KO. c, Representative flow cytometry plots of intracellular cytokine staining from TMBP cells (control and Ets1-KO) in vitro at 1 day post restimulation; grey, isotype control; lilac, control; green, KO. Right, quantification of the percentages. n = 5 independent experiments for control; n = 4 Ets1-KO. d, qPCR validation of selected upregulated genes in the Ets1-KO cells driving the upregulated pathways. n = 3 samples per group. e, Western blot showing the downregulation of ETS1 protein levels in the KO cells. n = 1. f, Experimental design of the bulk RNAseq experiment of Ets1-KO and control TMBP cells from the spleen of co-transferred animals. g, Volcano plot of the RNAseq results of Ets1-KO cells compared to control TMBP cells in the spleen, lines indicate adjusted p-value = 0.05 and log2(Fold Change) = ±3 standard deviations of the sample. Blue, genes downregulated in the Ets1-KO compared to control in parenchyma (“Ets1-KO downregulated in parenchyma”); red, genes upregulated in the Ets1-KO compared to control in parenchyma (“Ets1-KO upregulated in parenchyma”). Control FACS datapoints for in vitro surface (b) and intracellular stainings (c) are the same for all KOs, which were analysed in parallel (cf. Extended Data Figs. 4, 5 and 6). a,b,c, two-way ANOVA (a, F = 0.3755, P ns; b, F = 1.066, P ns; c, F = 0.2231, P ns) with multiple comparisons with the two-stage linear step-up procedure of Benjamini, Krieger and Yekuteli. d, multiple paired t-tests with two-stage linear step-up procedure of Benjamin, Krieger and Yekutieli multiple testing correction. Figures show mean ± s.d, P > 0.05 ns (non-significant), P < 0.05 *, P < 0.01 **, P < 0.001 ***, P < 0.0001 ****. Source data

In addition to the genes that facilitate CD4^+^ T cell entry to the CNS, our screen also detected five essential regulators, the loss of which resulted in enhanced T cell trafficking to the spinal cord meninges and parenchyma. The top-ranked hit among these brakes of endogenous CNS migration was the transcriptional regulator ETS1, which regulates differentiation, survival and proliferation of lymphoid cells^36^, and limits pathogenic T cell responses in atopic and autoimmune reactions^37,38^. To validate the role of ETS1 in T cell migration into the CNS, we again used CRISPR editing to delete this gene in T_MBP_ cells and then co-transferred *Ets1*-KO and control cells into rats and assessed their trafficking. This confirmed that *Ets1* deletion results in increased trafficking to both meninges and parenchyma (Fig. 6a,b). Subsequent transcriptional analysis of *Ets1*-KO CD4^+^ T cells isolated from the CNS compartment showed that these cells were likely to be more responsive to cytokine signals, and showed greater expression of genes encoding proinflammatory and cytotoxic mediators including IL-17, TNFRSF9, NKG7 and PRF1 (Fig. 6c–f), the expression levels of some of which were also increased in human *Ets1*-KO CD4^+^ T cells (Fig. 6g,h). In rats, this hyperresponsive and proinflammatory transcriptional phenotype is likely acquired as T cells traffic to the CNS as *Ets1*-KO CD4^+^ T cells isolated from the spleen showed no such transcriptional alterations and *Ets1*-KO CD4^+^ T cells before transfer did not display changes in the expression of activation molecules or the cytokines IFN-γ and IL-17A (Extended Data Fig. 7). The findings that ETS1 appears to restrict both the trafficking of T cells to the CNS and their proinflammatory and possibly cytotoxic actions in the target tissue makes this transcriptional regulator an interesting target for therapeutic applications.

### Essential regulators are associated with the CNS migration propensity of T cells in multiple sclerosis

**Fig. 7:**
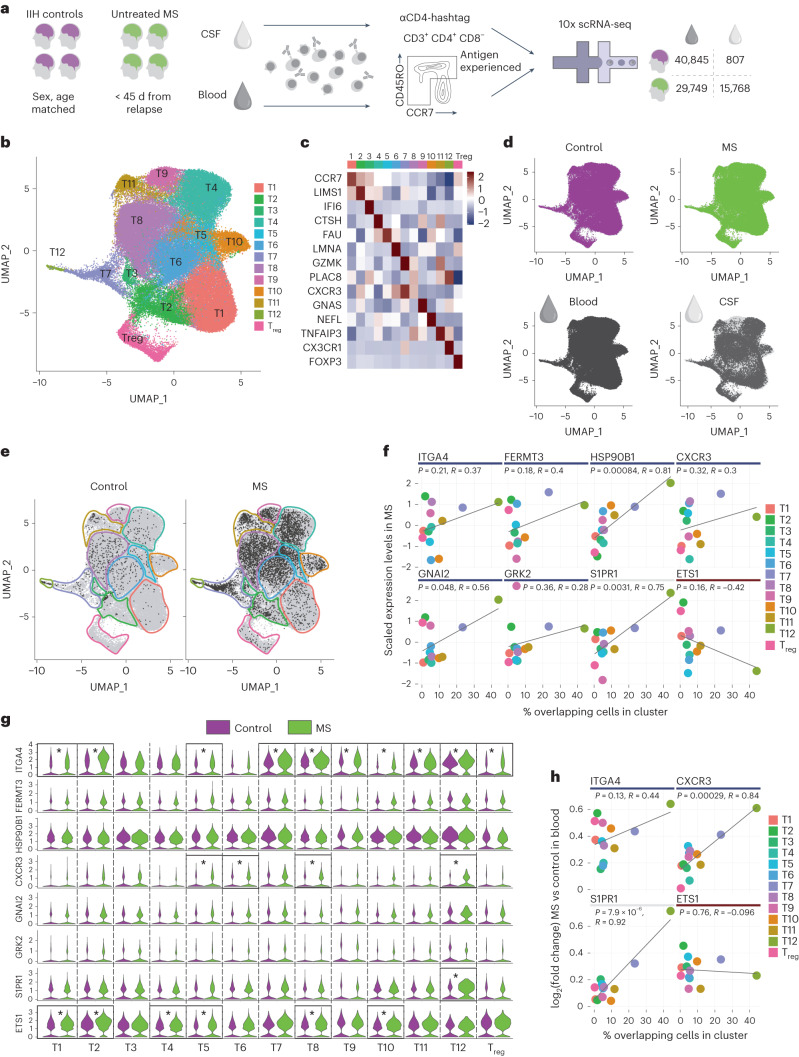
Expression of essential regulators of migration in T cells from participants with multiple sclerosis. **a**, Single-cell RNA-seq (scRNA-seq) experimental design. Sorted CD4^+^ T cells from the blood and whole CSF of untreated participants with MS, or sex-matched and age-matched controls, were collected and analyzed. **b**, CD4^+^ T cell clusters. **c**, Cluster-defining genes. **d**, Distribution of control or MS samples, and of blood or CSF samples, across the clusters. **e**, Overlapping cells in control and MS, across clusters, defined as cells whose TCR was found in blood and CSF of the same participant. **f**, Correlations between the percentage of overlapping cells per cluster and the relative gene expression level of essential regulators in this cluster in MS blood. **g**, Violin plots of the candidate genes expression level across clusters, comparing control (lilac) and MS (green) cell samples from blood. Asterisks indicate significance; adjusted *P* value < 0.05 and absolute log_2_(fold change) > 3 times the standard deviation of the sample. **h**, Correlations between the percentage of overlapping cells per cluster and the log_2_(fold change) of gene expression in this cluster between MS and control blood, for those essential regulators that show differences in expression levels between MS compared to control blood as in **g**. **f**,**h**, *R* value and *P* values correspond to a Pearson correlation. **g**, Asterisks correspond to adjusted *P* values < 0.05 comparing MS to control blood.

**Extended Data Fig. 8:**
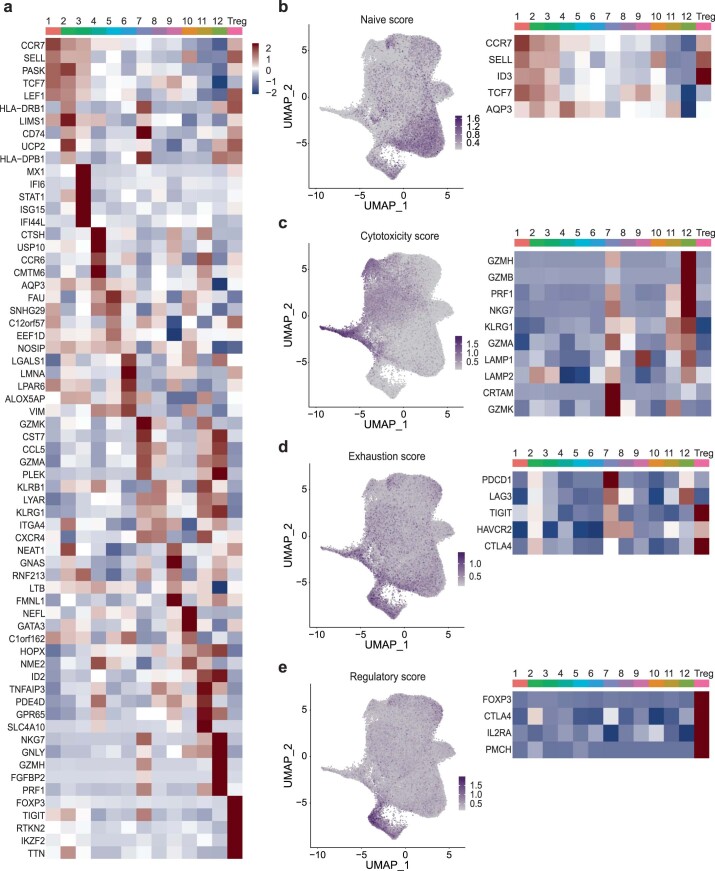
Molecular characterization of CD4 + T cell clusters. a, Heatmap showing the relative expression levels of the top five differentially regulated genes per cluster. b-e, Naïve (b), cytotoxicity (c), exhaustion (d) and regulatory (e) T cell marker gene expression, across cells (left) and clusters (right).

**Extended Data Fig. 9:**
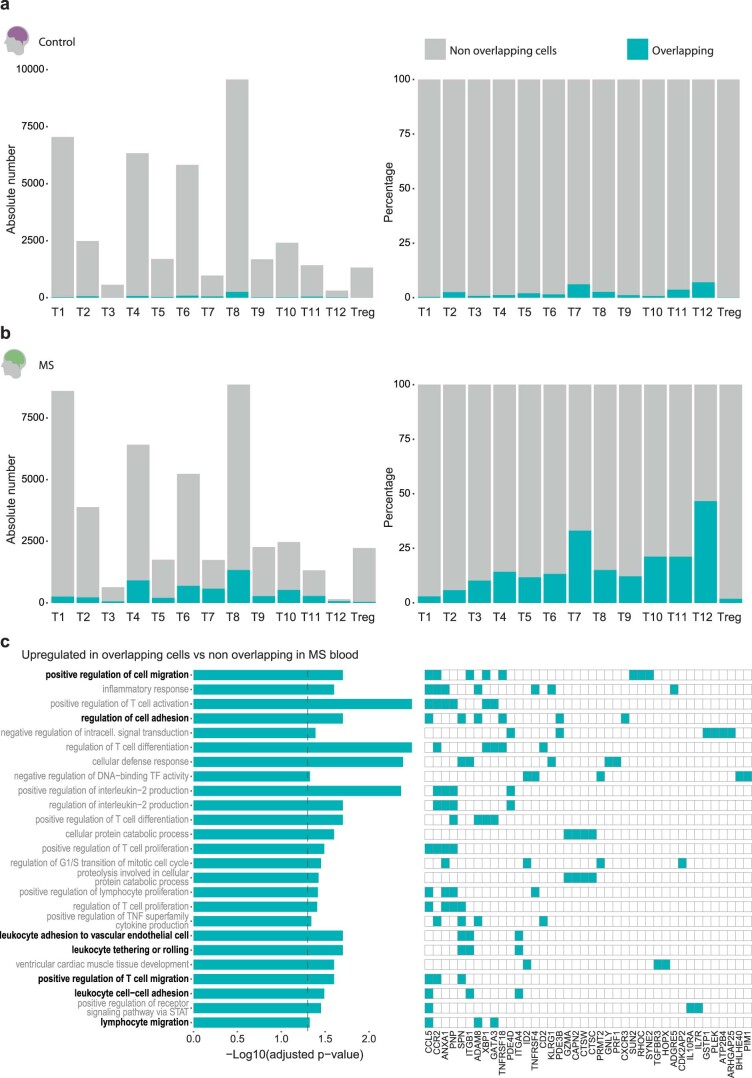
Differentially regulated pathways in T cell clones overlapping between CSF and blood in patients with MS. a, b, Absolute (left) and relative (right) numbers of overlapping CD4 + T cells (defined as cells belonging to a clone whose TCR was found in blood and CSF cells of the same donor) across clusters in controls (a) and in patients with MS (b). c, Pathway analysis of the top upregulated pathways in overlapping CD4 + T cells in the blood of patients with MS compared to non overlapping cells in the same compartment, left panel shows differentially regulated pathways and right panel shows the top differential regulated genes in these pathways. In bold, pathways related to migration. Source data

**Extended Data Fig. 10:**
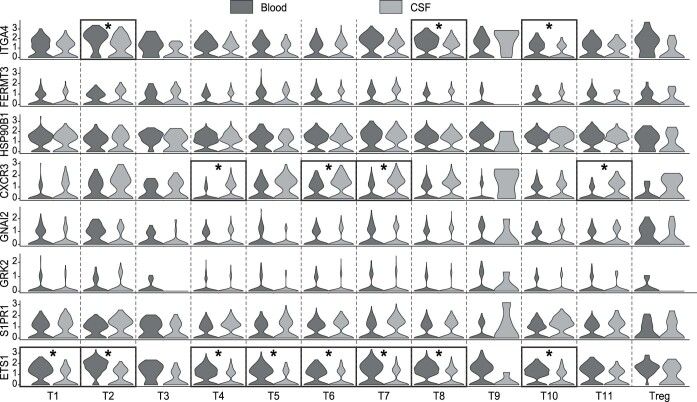
Expression pattern of essential regulators in human CD4 + T cells from blood and CSF. Violin plots comparing selected gene expression levels between T cells (analysed per cluster) from blood (dark grey) and CSF (light grey) in patients with MS for those CD4 + T cell clones whose TCR sequences were detected in blood and CSF. Stars indicate significance as per adjusted P < 0.05 and absolute log2(Fold Change) > 3 times the standard deviation of the sample. Starts indicate adjusted p-value < 0.05 when comparing MS CSF to blood.

To further assess the translational relevance of our findings, we performed single-cell transcriptomic analysis of 70,594 antigen-experienced CD4^+^ T cells isolated from the blood and 16,575 such cells isolated from the cerebrospinal fluid (CSF) of four untreated participants with MS and four control participants who had been diagnosed with idiopathic intracranial hypertension (IIH; Fig. 7a, for details, see Methods). Bioinformatic analysis revealed 12 distinct CD4^+^ helper T cell clusters (here termed T1 to T12) that differed in their level of activation, cytotoxicity and exhaustion, as well as a cluster of regulatory T (T_reg_) cells defined by FOXP3 expression (Fig. 7b,c and Extended Data Fig. 8). All T cell clusters were present in participants with MS and controls and all except for T12 were present in both blood and CSF (Fig. 7d and Methods). To identify those T cell clusters in the blood of participants with MS that are most likely to migrate to the CNS, we used their T cell antigen receptor (TCR) sequences as tags to determine the proportion of T cell clones in each cluster that were also found in the CSF compartment of the same participant (Fig. 7e). In line with the view that these ‘overlapping’ T cell clones have a higher propensity for CNS migration, a differential gene expression analysis showed that overlapping cells upregulate pathways associated with cell adhesion and cell migration (Extended Data Fig. 9). We then investigated whether this ‘CNS migration propensity’ of a CD4^+^ T cell cluster correlates with the expression pattern of the key components of the functional modules of CNS migration we identified in the EAE model and found that it did. HSP90B1, GNAI2 and S1PR1 expression levels in CD4^+^ T cell clusters from participants with MS were significantly correlated with their likelihood of migration into the CNS, while the expression level of ETS1, which limits CNS migration of T cells in rats, showed a trend toward a negative correlation with migration propensity (Fig. 7f). The expression of most of these regulators did not shift markedly after CNS entry as their expression (except for ITGA4, CXCR3 and ETS1) was comparable in T cell clones that were present both in the blood and CNS compartment (Extended Data Fig. 10). Finally, we asked whether the expression of the key components of the functional modules of CNS migration differed between T cells isolated from the blood of participants with MS and controls. We found that FERMT3, HSP90B1, GNAI2 and GRK2 were similarly expressed in CD4^+^ T cell clusters from participants with MS and controls, while the expression levels of ITGA4, CXCR3, S1PR1 and ETS1 were significantly higher in some of the T cell clusters from participants with MS (Fig. 7g). Notably, the differences in expression level of these key regulatory genes between cells from participants with MS and controls were greater in clusters with a higher migration propensity, again except for the negative regulator ETS1 (Fig. 7h). Taken together, our single-cell transcriptomic analysis of human helper T cells demonstrates that the essential regulators of T cell migration we identified in the rodent MS model are present in a sizable fraction of CD4^+^ T cells in the blood of participants with MS where their expression correlates with the capacity to enter the CNS.

## Discussion

Here we present the results of a genome-wide CRISPR screen aiming to identify the key molecules that regulate the critical initial step in the formation of the MS lesion: the infiltration of autoreactive CD4^+^ T cells from the blood to the CNS. We reveal the molecules that act as essential brakes and facilitators of the CNS transmigration of T cells, outline the modules that mediate their functional impact and show that their key molecular interactions are conserved across species. Our data also form the foundation for future studies that extend these findings, for example, by looking at the role of paracrine or systemic signals emitted by the cells in question (for example, cytokines, chemokines or matrix metalloproteases released by the autoreactive T cells), or that search for functionally important but molecularly redundant regulatory mechanisms, neither of which can be assessed using a CRISPR screen. Further limitations of the CRISPR screen approach include that molecules can be missed if they only regulate the trafficking of a smaller subset of the studied cell population. Finally, false positive results may arise if a gene regulates a functional property such as T cell activation that is likely required for subsequent CNS migration. We believe this is unlikely to be the case for the essential regulators we identify here because a comparison between the activated and expanded T cells at the end of the culture period with the plasmid library showed a depletion of genes associated with T cell activation and proliferation but did not show a marked regulation of the essential mediators of migration (Extended Data Fig. 1). Despite its limitations, the CRISPR-based screening of MS models that we introduce here represents a highly versatile approach that can be easily adapted to interrogate the subsequent steps of T cell-mediated CNS pathology or could be leveraged to resolve the migration of other immune cells with critical contributions to MS pathology such as CD8^+^ T cells, B cells or monocytes.

What did we learn from this unbiased and comprehensive characterization of the essential molecular signals governing autoreactive T cell entry to the CNS? First, that remarkably few molecules are essential for T cell migration into the CNS, and that the majority of the most potent mediators naturally cluster in three functional modules: one centered around the adhesion molecule α4-integrin, another around the chemokine receptor CXCR3 and the final one involving the S1PR1–GRK2 axis. While some of these molecules have been implicated in T cell migration in general, or even in MS, before, this unbiased analysis reveals all nonredundant targets among their transcriptional regulators, chaperones and binding partners as well as their intracellular signaling streams. Alongside, many previously suspected candidate regulators did not appear as ‘essential’ in our screen. These included the CCR5, CCR6 and CCR7 chemokine receptors^7,39,40^, the adhesion molecules P-selectin^41^, Ninjurin-1 (refs. ^5,42^), MCAM^43^, DICAM^44^, the ALCAM ligand CD6 (refs. ^45,46^) and LFA-1 (ref. ^47^). This raises interesting questions around whether these mediators are functionally redundant, or whether they are only required for a subpopulation of CD4^+^ T cells, as for LFA-1, MCAM and DICAM, which are primarily important for the migration of the T_H_17 subset of helper T cells^43,44,48^.

A second important finding of our screen is the presence of endogenous ‘brakes’ of T cell migration, such as the transcription factor ETS1, which appears to limit the responsiveness of T cells to a range of immunological signals. Notably, ETS1 also appears to restrict the expression of a number of cytotoxic mediators making the induction of this transcriptional regulator an attractive strategy in autoimmune conditions. Furthermore, inhibition of ETS1 might be of therapeutic interest in the context of brain cancer or neurodegenerative diseases in which insufficient immune responses in the CNS can contribute to pathology^49,50^.

Importantly, we showed that the essential modules we identified in the rat EAE model have high likelihood of relevance in participants with MS. Two of the major clinical strategies that limit CNS infiltration of T cells in people with MS, blocking α4-integrin and interfering with the S1PR1 receptor, also emerge as central functional hubs in our genome-wide screen. While established S1PR1 modulators induce receptor internalization, we show here that interfering with receptor desensitization via GRK2 is a distinct regulatory mechanism that is active in human T cells and has an even more potent effect on CNS migration, at least in our rodent MS model. Interestingly, a previous study reported reduced GRK2 protein levels in peripheral blood mononuclear cells (PBMCs) isolated from people with relapsing–remitting and secondary progressive MS suggesting an altered regulation of S1PR1 internalization^31^. We further show that the expression pattern of the essential regulators we identified in the EAE model reflects the propensity of defined T cell clusters from individuals with MS to reach the CNS. Alongside, the expression of several of these regulators, most prominently of α4-integrin and CXCR3, is specifically higher in CNS migration-prone T cell clusters in individuals with MS compared to controls. These data extend the results from previous population-based analyses of T cells in people with MS^51,52^ and provide a molecular explanation for enhanced T cell entry to the CNS in MS. Taken together, our study thus helps to define the essential molecules and modules that govern CD4^+^ T cell trafficking to the CNS and demonstrates their regulated expression in individuals with MS.

## Methods

### Contact for reagent and resource sharing

Further information and requests for resources and reagents should be directed to and will be fulfilled by N.K. Plasmids generated in this study are available upon reasonable request.

### Experimental model and participant details

#### Plasmids

All primer sequences are listed in Supplementary Table 3.

The pMSCV-Cas9-EGFP vector was constructed as follows. First, a Cas9-p2a-EGFP construct was PCR amplified from the Lenti-Cas9-EGFP plasmid obtained from Addgene (63592). Then, the PCR product was assembled into an EcoRI + XhoI digested pMSCV-neo (Takara Clontech) vector by using Gibson Assembly Master Mix (NEB). The gRNA expression vector MSCV-pU6-(BbsI)-CcdB-(BbsI)-Pgk-Puro-T2A-BFP was obtained from Addgene (86457). The MSCV-v2-U6-(BbsI)-Pgk-Puro-T2A-GFP (with v2 improved scaffold) vector was generated as follows: first, the pU6-Pgk-Puro-T2A construct was PCR amplified from pKLV2-U6gRNA5(BbsI)-PGKpuro2ABFP-W (Addgene, 67974), and the EGFP construct was PCR amplified from pMSCV-Cas9-EGFP; then, the PCR product was assembled into the SalI + XhoI digested pMSCV-neo vector using the Gibson Assembly Master Mix. The pMSCV-v2-U6-(BbsI)-Pgk-Puro-T2A-BFP (with v2 improved scaffold) vector was generated as follows: the pU6-Pgk-Puro-T2A-BFP construct was PCR amplified with overhangs for SalI + XhoI from pKLV2-U6gRNA5(BbsI)-PGKpuro2ABFP-W, then the PCR product was digested and ligated into SalI + XhoI digested pMSCV-neo vector with Quick Ligase (NEB).

#### Animals

Lewis rats were purchased from Charles River (LEW/Crl) or Janvier (LEW/OrlRj) and bred in the Core Facility Animal Models of the Biomedical Center, LMU. All animal experiments and their care were carried out in accordance with the regulations of the applicable animal welfare acts and protocols were approved by the responsible regulatory authority (Regierung von Oberbayern). All animals had free access to food and water. Animals were kept at room temperature 22 ± 2 °C, humidity 55% ± 10% with a light–dark cycle, 12 h/12 h (6:30–18:30). Male and female Lewis rats between 5 and 20 weeks old were used for the experiments. The animals were allocated randomly for experimental groups.

#### Generation of Cas9-expressing T_MBP_ cells

Cultures of T_MBP_ cells were established as previously described^53^. Briefly, a Lewis rat was immunized in the hind-limb flanks with 100 µg guinea pig MBP (purified in-house) emulsified with complete Freund’s adjuvant (Difco). Draining lymph nodes were collected 10 d after immunization and a single-cell suspension was prepared by passing through a metal strainer. The cells were then restimulated ex vivo with 10 µg ml^−1^ MBP either together with retrovirus producing GP + E86 (American Type Culture Collection (ATCC) packaging cells stably transfected with pMSCV-Cas9-EGFP, or without packaging cells, in complete DMEM supplemented with 1% rat serum. Two days later, TCGF (complete DMEM supplemented with 10% horse serum and 2% of phorbol myristate acetate (PMA)-stimulated EL4IL2 cell culture supernatant) was added to expand the number of T cells. After 4 d of expansion culture, the T cells were restimulated for 2 d with 50-Gy irradiated thymocytes in complete DMEM supplemented with 1% rat serum and 10 µg ml^−1^ MBP, which was followed by another round of expansion in TCGF for 4 d. This cycle of restimulation and expansion was repeated before some experiments. In addition, successfully transduced T cells were enriched by adding 400 µg ml^−1^ neomycin for 8 d from 4 d after the first stimulation and sorting for GFP^+^ cells with a BD FACSAria IIIu operated with FACSDiva. The antigen specificity of the cultured T cells was confirmed by a proliferation assay, as described previously^18^. Cells were frozen in a 10% dimethyl sulfoxide/90% FBS mixture at −80 °C, or stored in liquid nitrogen for long-term storage.

#### Study participants

CSF sampling was performed to confirm the diagnosis of relapsing–remitting MS according to the revised McDonald criteria for all (four) participants included in the study, who had encountered an MS relapse in the 45 d before lumbar puncture and had not received any disease-modifying treatments (one participant was treated with high-dose steroids 24 d before sampling). CSF and blood samples from four sex-matched and age-matched individuals diagnosed with IIH were included as the control group. Participant samples were collected at the Institute of Clinical Neuroimmunology at the LMU Klinikum Munich, Germany. Recruitment of participants took place from August 2020 to January 2021. Collection of blood and CSF was approved by the local ethics committees of the LMU, Munich (ethical vote no. 163-16). Written informed consent was obtained from all participants according to the Declaration of Helsinki.

PBMCs of four healthy donors used for CRISPR gene editing were derived from leukoreduction system chambers provided by the Department of Transfusion Medicine at the LMU Klinikum Munich, Germany (ethical vote LMU no. 18-821).

### Method details

#### Genome-wide rat guide RNA library construction

A list of sgRNAs targeting genes and miRNAs in the rat genome was kindly provided by the Functional Genomics Consortium of The Broad Institute, Massachusetts, USA. All sgRNA sequences that were selected, and the library cloning primers, as well as protocols, are listed in Supplementary Table 3.

For the genome-wide library, in nearly all cases four sgRNA per gene or miRNA were selected; in some cases, for example, for miRNAs only fewer unique sgRNAs were available. A total of 87,690 oligonucleotides were purchased from Twist Bioscience, each as a 79-mer with a sequence of 5′- GCAGATGGCTCTTTGTCCTAGACATCGAAGACAACACCGN_20_GTTTTAGTCTTCTCGTCGCC-3′, N_20_ indicating the sgRNA sequence. Library cloning was performed as previously described^54^ with minor modifications to the primer sequences and protocol (Supplementary Table 3). Briefly, oligonucleotide pools were dissolved in TE buffer at a 10 ng μl^−1^ stock concentration, then the single-stranded oligonucleotides (1 ng) were PCR amplified for 10 cycles with Q5 High Fidelity DNA Polymerase (NEB) using Oligo_Amp_F and Oligo_Amp_R primers to generate double-stranded DNAs. A total of 24 reactions were pooled. The PCR products were purified with the Nucleotide Removal Kit (Qiagen). Amplified double-stranded DNAs were digested with FastDigest BpiI (BbsI: Thermo Fisher) for 2 h at 37 °C in a total of 20 reactions, and then purified with the Nucleotide Removal Kit. Ligation was performed with a T4 DNA Ligase (NEB) using a 3-ng insert and 40 ng BpiI-digested MSCV-pU6-(BbsI)-CcdB-(BbsI)-Pgk-Puro-T2A-BFP for 16 h at 16 °C per reaction in a total of 30 reactions. The ligated product was cleaned with a PCR Purification Kit (Qiagen) and the concentration was measured with Qubit 4 (Thermo Fisher). Next, 10 ng of the ligated product was transformed into 50 μl of NEB Stable Competent cells in a total of 45 reactions and incubated at 30 °C overnight. A library representation above 100× was confirmed by plating transformed competent cells in serial dilutions. The plasmid DNA was prepared with an Endofree Plasmid Maxi Kit (Qiagen). For the validation library, six sgRNAs were used per gene whenever possible. An oligonucleotide pool containing 12,000 oligonucleotides was purchased from Twist Bioscience and plasmid DNA was prepared similarly to the genome-wide library.

#### Gene editing of T_MBP_ cells

For viral transduction of CRISPR sgRNAs, 1.2 × 10^6^ HEK293T cells (ATCC) in complete DMEM with 10% FBS were plated into a 10-cm diameter culture dish 18–24 h before transfection. For the transfection, 6 µg pMSCV retroviral plasmid and 3.5 µg pCL-Eco packaging vector were preincubated in 500 µl of complete DMEM at room temperature for 15 min before mixing with 500 µl of 80 µg ml^−1^ PEI max (Polysciences) in complete DMEM. After 20 min, the solution was added dropwise to HEK293T cells. The cells were cultured in 5% CO_2_ at 37 °C for 24 h and the medium was replaced with 8 ml complete DMEM + 10% FBS for detoxification. The cells were cultured in 10% CO_2_ at 37 °C, and retrovirus-containing supernatant was collected at 48 h and 72 h after transfection. The supernatant was then passed through a 0.45-µm filter to remove debris and virus was concentrated using Amicon Ultra-15 Centrifugal Filter Units (Sigma). For this, 14 ml supernatant was centrifuged at 4,000*g* for 20 min at room temperature. The concentrated virus was used immediately for transduction of rat T cells.

For transduction, freshly restimulated T cells were resuspended in DMEM with 25 mM HEPES and then enriched by Nycoprep gradient centrifugation at 800*g* for 10 min at room temperature. The T cells were resuspended at concentration of 4 × 10^6^ per ml in TCGF with 8 µg ml^−1^ polybrene (Sigma) and this suspension was plated at 500 µl per well in 12-well plates. Finally, concentrated virus solution was added at 50 µl per well and plates were centrifuged at 2,000*g*, at room temperature for 90 min. TCGF was added at 1 ml per well and T cells were further cultured in 10% CO_2_ at 37 °C. T_MBP_ cells were transduced at a maximum MOI of 0.3 to prevent multiple integrations, and enough T cell numbers were kept at all times to ensure a minimum 1,000× coverage in vitro (1,000 T cells having the same sgRNA; 90 × 10^6^ cells for the genome-wide screen and 12 × 10^6^ cells for the validation screen). On the next day, puromycin was added at a final concentration of 0.5 µg ml^−1^ to select for transduced T cells. After 4 d of culture, the T cells were restimulated as described above for 2 d, before injection into recipient animals. For T cell transfer into animals, cell numbers injected were such to maintain a minimum coverage of 1,000× per replicate.

For candidate gene validation, CRISPR sgRNAs were introduced by ribonucleoprotein (RNP) electroporation into previously BFP or EGFP retrovirus transduced T_MBP_ cells. sgRNAs were designed by using the GPP sgRNA designer^55,56^ and synthesized by Integrated DNA Technologies. The Cas9 protein and sgRNA were electroporated into the T cells by using Amaxa 4D-Nucleofector System and P4 Primary Cell 4D-Nucleofector X Kit S (Lonza) according to the manufacturer’s instructions. Briefly, to prepare the transfection reagent, 0.75 µl of Alt-R CRISPR–Cas9 trans-activating crRNA (tracrRNA; 200 pmol µl^−1^) and 0.75 µl of Alt-R CRISPR–Cas9 crRNA (200 pmol µl^−1^; IDT) were mixed; the solution was then incubated at 95 °C for 5 min, decreasing to 70 °C at the rate of 0.5 °C s^−1^, at 70 °C for 1 min, then cooled to 22 °C. After adding 7.5 µg Alt-R S.p. HiFi Cas9 Nuclease V3 (IDT), the mixture was incubated at room temperature for 20 min. The master mix was prepared by mixing 18 µl of P4 primary solution, 4 µl of Supplement 1 and 1 µl of electroporation enhancer (stock solution of 100 µM; IDT). After washing with PBS, 2 × 10^6^ T cells were pelleted and resuspended in 21 µl of master mix. This cell suspension was mixed with the transfection reagent and transferred into the Nucleofection cuvette. Electroporation was performed using the pulse code CM137.

#### Tide assay

All single KO lines used in validation experiments were validated for the KO efficiency before the experiment (Supplementary Table 3). To assess the extent of genetic editing at DNA level for single sgRNAs, cells were lysed by lysis buffer (H_2_O with 100 µl ml^−1^ 1 M Tris, 10 µl ml^−1^ 0.5 M EDTA, 40 µl ml^−1^ 3 M NaCl, 5 µl ml^−1^ Tween 20) supplemented with 5 µl ml^−1^ Proteinase K for 15 min at 56 °C and 10 min at 95 °C followed by cooling on ice. Then, 350 µl isopropanol was added, incubated for 10 min at room temperature and centrifuged for 10 min at 16,000*g* at 4 °C. The pellet was washed by addition of 1 ml of 70% ethanol and 5 min centrifugation at 16,000*g* at 4 °C. After removal of liquid, the pellet was dried for 10 min at 56 °C and resuspended in 50 µl water. PCR amplification of the modified DNA sequence was performed with specific primers for each target using 2× Optima PCR HotStart Polymerase (FastGene). PCR products were run on a 1% agarose gel and amplicons of the expected size were purified using the Wizard SV Gel and PCR Clean-Up System (Promega). For human T cells, genomic DNA was isolated with the DNeasy Blood and Tissue Kit (Qiagen) and the modified DNA region was amplified with Q5 High Fidelity DNA Polymerase (NEB). The samples were submitted to Sanger Sequencing (Sequencing service, LMU Biozentrum). The INDELs and KO efficiency were assessed by the ICE v2 software tool^57^.

#### Adoptive transfer experimental autoimmune encephalomyelitis and isolation of T_MBP_ cells

EAE was induced in rats by intravenous adoptive transfer of in vitro activated T_MBP_ cells. Following transfer, body weight and the EAE score of the rats were monitored daily. The EAE score was evaluated as follows: 0, no clinical signs; 0.5, partial tail weakness; 1, tail paralysis; 1.5, gait instability or impaired righting ability; 2, hind-limb paresis; 2.5, hind-limb paresis with dragging of one foot; 3, total hind-limb paralysis. The number of transferred T cells was about 12 × 10^6^ for screening (a minimum of eight animals were pooled for a single genome-wide replicate and a minimum of six animals were pooled per validation screen replicate), a mixture of 3 × 10^6^ control T cells and 3 × 10^6^ KO T cells were transferred for evaluating their migration to the CNS, and 1 × 10^6^ T cells were transferred for the assessment of the clinical course.

For screening and in vivo migration analysis, the animals were euthanized on day three after T cell transfer, when animals showed first clinical symptoms, such as body weight loss and/or a mild clinical score (<1). Blood was drawn by heart puncture into a heparinized syringe. Spleen, parathymic lymph nodes, leptomeninges and parenchyma of the spinal cord were dissected and homogenized by passing through a metal strainer. Lymphocytes were isolated from blood by a Nycoprep gradient. First, the blood was diluted with an equal volume of PBS and overlaid onto Nycoprep. After centrifugation at 800*g*, room temperature for 30 min with mild acceleration and brake, lymphocytes were collected from the interface. For spleen, erythrocytes were removed by treating with ACK buffer for 3 min on ice and CD4^+^ T cells were enriched using the EasySep Rat CD4^+^ T Cell Isolation Kit (StemCell technologies), before purification by sorting. From the spinal cord parenchyma, the lymphocytes were isolated using a 30%/64% Percoll gradient and centrifugation at 1,200*g*, room temperature for 30 min with mild acceleration and brake. Lymphocytes were collected from the interface.

#### Flow cytometry

For the CRISPR screens, BFP^+^ T_MBP_ cells were sorted from spleen only (genome-wide screen) or from all four tissues (validation screen) using a FACS Aria III (BD) or FACS Fusion (BD) with FACSDiva at the Flow Cytometry Core Facility of the Biomedical Center, LMU. Cells were sorted to achieve an sgRNA coverage of >100× (the same sgRNA being present in at least 100 sorted T cells). The expression of cell surface molecules was measured by flow cytometry after antibody labeling. T cells were incubated for 30 min on ice with the primary antibody diluted in FACS buffer (PBS with 1% rat serum and 0.05% NaN3). After washing three times with FACS buffer, the cells were incubated with a fluorochrome-conjugated secondary antibody in FACS buffer for 30–45 min on ice. The following antibodies were used with a 1:100 dilution unless stated otherwise: Mouse IgG1 Isotype control (Sigma), Armenian hamster IgG2 Isotype control (BD), mouse anti-rat CD49d (Thermo Fisher), mouse anti-rat CD11a (BioLegend), mouse anti-rat CD18 (Thermo Fisher), mouse anti-rat TCRβ (BD), mouse anti-rat CD25 (Thermo Fisher), mouse anti-rat CD134 (Thermo Fisher), Armenian hamster anti-rat CD29 (BioLegend), APC conjugated donkey anti-mouse IgG (Jackson ImmunoResearch, 1:1,000 dilution), APC conjugated goat anti-Armenian hamster IgG (Jackson ImmunoResearch, 1:1,000 dilution) and AF647 conjugated goat anti-mouse IgG (SouthernBiotech, 1:1,000 dilution). Cells were then washed once in FACS buffer and once with PBS, then resuspended in PBS and analyzed by flow cytometry. For intracellular staining, T cells were incubated with 5 µM Brefeldin (Sigma Aldrich) in complete DMEM supplemented with 1% rat serum for 5 h at 37 °C and fixed with 2% paraformaldehyde for 20 min on ice. The T cells were stored in PBS at 4 °C until intracellular staining. The staining was performed similarly to the surface staining with the following antibodies diluted in permeabilization buffer (BioLegend): mouse IgG1 Isotype control (Sigma), PE conjugated rat IgG1 isotype control (BD), mouse anti-rat IFN-γ (eBioscience), PE conjugated rat anti-mouse/rat IL-17A antibody (BD) and APC conjugated donkey anti-mouse IgG (Jackson ImmunoResearch). For assessment of in vivo migration and ex vivo cell surface molecule detection, BFP^+^ T_MBP_ cells and EGFP^+^ T_MBP_ cells were analyzed using a FACS VERSE (BD) with FACS Suite, LSRFortessa (BD) flow cytometer with FACSDiva or Cytoflex S with CytExpert (Beckman Coulter). For in vitro surface and intracellular staining, cells were analyzed with Cytoflex S.

For in vivo migration assays, the KO/control ratio based on the numbers of BFP^+^ or EGFP^+^ cells in the lymphocyte gate was calculated for all tissue samples and normalized to the blood ratio of the same animal (tissue KO cell number/control cell number ratio divided by the blood KO/control ratio in the same animal). Thus, the migration phenotype for the gene KO was always compared to the migration control T_MBP_ cells within that animal to correct for inter-animal and inter-experiment variability.

#### Next-generation sequencing sgRNA library preparation

Genomic DNA from lymphocytes or sorted T_MBP_ cells was isolated with the DNeasy Blood and Tissue Kit (Qiagen). A one-step PCR amplification was performed with Q5 High Fidelity DNA Polymerase by using 2.5 µg of genomic DNA per reaction with Fwd-Lib (mix of eight staggered primers) and Rev-Lib (consists of 8 bp of unique barcode) primers for a total of 24 cycles. Illumina adaptors were introduced together with the amplification primers. All primer sequences are listed in Supplementary Table 3. The amplified DNA amplicons were purified with SPRIselect (Beckman Coulter) with a ratio of 1:0.8 (DNA to beads) and eluted in nuclease-free water. The presence of ~250-bp DNA amplicons was confirmed, and the concentration was measured with Agilent Bioanalyzer on DNA 1000 chips (5067-1504). Library samples were sent to The Laboratory for Functional Genome Analysis (LAFUGA) at the Gene Center Munich for sequencing single-end 50 bp on a HiSeq 1500.

#### qPCR and 3′ bulk mRNA sequencing

All primer sequences are listed in Supplementary Table 3.

Total RNA from cells was isolated with either an RNeasy Plus Mini (Qiagen) or a Micro (Qiagen; for less than 100,000 cells) kit according to the manufacturer’s protocol. For qPCR, cDNA was synthesized using the RevertAid H Minus First Strand cDNA synthesis kit (Thermo Fisher) with 100–500 ng total RNA and Oligo (dt) primers and assays were performed on the Bio-Rad CFX Connect Real-Time PCR system using SsoAdvanced Universal SYBR Green Supermix (Bio-Rad). The β-actin housekeeping gene was used to normalize the variability in expression level. All qPCR reactions were run in duplicates. Results were quantified using the ΔΔCt method. For 3′ bulk mRNA-seq, the library was prepared from total RNA using the Collibri 3′ mRNA Library Prep Kits for Illumina Systems (Thermo Fisher). Amplification of transcripts was confirmed with an Agilent Bioanalyzer on DNA 1000 chips and sent to LAFUGA (Gene Center, LMU Munich) for single-end 50-bp sequencing on a HiSeq 1500.

#### Western blotting

For assessment of Phospho-ERK levels after stimulation of S1PR1 signaling, T cells were incubated in medium consisting of 1 µM S1P (Tocris) for 10 min at 37 °C. For all western blot analyses, T_MBP_ cells were washed twice with PBS and lysed with RIPA buffer (Thermo Fisher) including inhibitors of proteases (Sigma) and phosphatases (Sigma). Pierce Bovine Serum Albumin Standard Pre-Diluted Set Kit (Thermo Fisher) or Pierce Rapid Gold BCA Protein Assay Kit (Thermo Fisher) was used to calculate the protein concentration of samples. Lysates were boiled at 95 °C for 5 min in a mix with Tris-Glycine SDS sample buffer (Thermo Fisher) and reducing agent (Thermo Fisher). Lysates were resolved in 4–12% Tris-Glycine gels (Thermo Fisher) for protein separation and transferred to PVDF membrane (Millipore) using Mini Gel Tank and Blot Module (Thermo Fisher). Anti-GRK2 (CST, 1:1,000 dilution), anti-phospho-p44/42 MAPK (ERK1/ERK2; Thr202/Tyr204; CST, 1:1,000 dilution), anti-p44/p42 MAPK (ERK1/ERK2; CST, 1:1,000 dilution), anti-ETS1 (CST, 1:1,000 dilution) and horseradish peroxidase-conjugated β-actin (Santa Cruz, 1:100,000 dilution) primary antibodies were incubated on the membrane overnight at 4 °C in 5% BSA-TBST buffer, followed by incubation with a horseradish peroxidase-conjugated secondary rabbit antibody (Santa Cruz, 1:10,000 dilution) at room temperature for 2 h. Blots were imaged using the LiCor Odyssey Fc system after treatment with ECL Western Blotting-Substrate (Thermo Fisher) or SuperSignal West Femto Maximum Sensitivity Substrate (Thermo Fisher) and analyzed with Image Studio Lite Software (LiCor)

#### Transwell chemotaxis assay

Chemotaxis assays were performed using a 96-well transwell chamber with a 5-μm pore size (Corning). T cells were resuspended in complete DMEM supplemented with 1% rat serum. Control-BFP^+^ and KO-EGFP^+^ cells were counted and mixed at a ratio of 1:1. Each upper insert received 0.2 × 10^6^ T cells in 75 μl medium. To the lower compartment, 235 μl of complete DMEM supplemented with 1% rat serum with or without chemotactic stimuli (30 ng ml^−1^ CXCL10, PeproTech or CCL5, PeproTech) was added. The chemotaxis plates were centrifuged (400*g*, 1 min) and incubated at 37 °C with 10% CO_2_ for 5 h. After incubation, migrated cells in the lower chamber were analyzed by flow cytometry using an LSRFortessa (BD) with FACSDiva or CytoFlex S with CytExpert (Beckman Coulter). For analysis and quantification, the percentage of cells detected in the lower chamber was normalized to input values. Then, the KO/control ratio was calculated for all conditions.

#### Intravital imaging of the spinal cord leptomeninges

Two or three days after intravenous co-transfer of 1 × 10^6^ BFP^+^ control and 1 × 10^6^ EGFP^+^ *Grk2*-KO T cells, intravital imaging within the spinal cord leptomeninges was performed as previously described^6^. The animal was anesthetized by intramuscular injection of MMF (2 mg per kg body weight midazolam, 150 µg per kg body weight medetomidine and 5 µg per kg body weight fentanyl), and a tracheotomy was performed to allow mechanical ventilation with 1.5–2.0% isoflurane in air. The body temperature of the animal was maintained by a heat pad placed underneath. Furthermore, a catheter was inserted into the tail vein to allow intravenous injection of Texas Red-conjugated 70-kDa dextran (100 µg) to visualize blood plasma during the imaging. To allow imaging, a laminectomy was performed at the dorsal part of Th12/Th13. For this, the skin was opened with a midline incision of 3 cm and the paravertebral musculature on the spine was removed. Then the animal was fixed in a custom-made fixation device, which provides stability by pushing with three pins from one side to the spine. The dorsal part of the central spine disc was removed after cutting both sides using a dental drill. The dura was then removed. To avoid artifacts due to breathing, the animal was slightly lifted before starting imaging. Time-lapse images were acquired with a Leica SP8 microscope using a water-immersion ×25 objective lens (numerical aperture of 1.00, working distance of 2.6 mm). For excitation of BFP and EGFP, a pulsed laser from an InSight DS+ Single (Spectra Physics) was adjusted to 840 nm, and fluorescence signals were first separated with a beam splitter (BS560). Signals of shorter wavelength were again split by BS505 and detected after the band-pass filters HC405/150 (BFP) and ET525/50 (EGFP). Signals of a longer wavelength were again separated by beam splitter RSR620 and detected after the band-pass filter BP585/40 (Texas Red). Images were acquired from a field of approximately 440 µm × 440 µm with a resolution of 512 × 512 pixels and an approximate 100 µm *z*-stack, with an interval of 2–3 µm.

The images were processed by Fiji. First, a Gaussian blur filter (cutoff of 1 pixel) was used, followed by maximum *z*-projection. When necessary, bleed-through liner subtraction was applied. Finally, signal intensity was adjusted by linearly adjusting brightness and contrast. For tracking of the cells, the Manual Tracking Plugin of Fiji was used to obtain coordinates, which were used to calculate migration speed and distance of the cells in Excel together with information from imaging such as time and pixel resolution. Locations of cells were analyzed by the Cell Counter Plugin of Fiji.

#### CRISPR editing of human CD4^+^ T cells

Cells derived from leukoreduction system chambers were diluted at a 1:5 ratio with PBS and added to a SepMate tube containing human Pancoll. During centrifugation (1,200*g* for 10 min at 4 °C), PBMCs were isolated by density gradient. CD4^+^ human T cells were enriched from PBMCs using EasySep Human CD4^+^ T Cell Isolation Kit (StemCell Technologies) according to the manufacturer’s protocol. CRISPR RNPs targeting control (NT), S1PR1–GRK2, ETS1, HSP90B1, CXCR3 and TBX21 were delivered into the CD4^+^ Human T cells by using Amaxa 4D-Nucleofector System and P2 Primary Cell 4D-Nucleofector X Kit S (Lonza) according to the manufacturer’s instructions using the pulse code EH100 (ref. ^58^). Briefly, per reaction, 0.375 µl of Alt-R CRISPR–Cas9 tracrRNA (200 pmol µl^−1^) and 0.375 µl of Alt-R CRISPR–Cas9 crRNA (200 pmol µl^−1^) were mixed and the solution was then incubated at 95 °C for 5 min, decreasing to 70 °C at the rate of 0.5 °C s^−1^, at 70 °C for 1 min, then allowed to cool to room temperature. Next, 5 µg Alt-R S.p. HiFi Cas9 Nuclease V3 (IDT) was mixed with crRNA:tracrRNA duplex and the mixture was incubated at room temperature for 20 min for RNP formation. After washing with PBS, 2 × 10^6^ T cells were pelleted and resuspended in 23 µl of P2 nucleofection buffer and mixed with RNP complex. Then, 0.8 µl of Alt-R Cas9 Electroporation Enhancer (stock solution of 100 µM; IDT) was added per reaction before the electroporation. RNP electroporated T cells were transferred to round-bottom 96-well plates (Corning) at a concentration of 1 × 10^5^ T cells per well in RPMI medium supplemented with Primocin (100 µg ml^−1^; Invivogen) and 10% charcoal-stripped FBS (Thermo Fisher). For stimulation of T cells, the medium was additionally supplemented with monoclonal anti-CD3 (1 µg ml^−1^; Thermo Fisher) and anti-CD28 (2 µg ml^−1^; Thermo Fisher) for 72 h at 37 °C in an incubator. T cells were then washed and further incubated with recombinant human IL-2 (10 ng ml^−1^; R&D systems) for up to 3 weeks in a 37 °C/5% CO_2_ incubator with medium change every 3 d. All crRNA sequences are listed in Supplementary Table 3. For ETS1, HSP90B1, CXCR3 and TBX21 editing, CD4^+^ T cells between day 7 and day 21 after isolation were used. T cells were washed with PBS and stained with anti-CD4-FITC (BioLegend), anti-CXCR3-PE (BioLegend), anti-CD49d-APC (BioLegend), anti-CD11a-PE (BioLegend) and anti-CD29-PE (BioLegend) antibodies at a concentration of 1:100 and with LIVE/DEAD Violet dye (Thermo Fisher) at a 1:1,000 concentration for 30 min in FACS buffer (PBS with 1 mM EDTA and 1% charcoal-stripped FBS) and analyzed by flow cytometry using CytoFlex S with CytExpert (Beckman Coulter).

#### S1PR1 internalization assay with human CD4^+^ T cells

Charcoal-stripped FBS was used in the medium to prevent unwanted S1PR1 receptor internalization. Human CD4^+^ T cells between day 6 and day 13 after isolation were used for the S1PR1 internalization assay. T cells were incubated in control medium (with PBS containing 4% fatty-acid-free BSA (Sigma) used to dissolve S1P as vehicle) or medium consisting of either 1 µM S1P (Tocris) or 1 nM fingolimod-P (FTY720 Phosphate, Biomol) for 90 min at 37 °C. T cells were then washed with PBS and stained with anti-CD4-FITC (BioLegend) and anti-S1PR1-eF660 (Thermo Fisher) at a concentration of 1:100 and with LIVE/DEAD Violet dye (Thermo Fisher) at 1:1,000 concentration for 30 min in FACS buffer (PBS with 1 mM EDTA and 1% charcoal-stripped FBS). S1PR1 expression in CD4^+^ T cells was assessed by flow cytometry using CytoFlex S with CytExpert (Beckman Coulter).

#### Collection and processing of human samples for single-cell transcriptomics

*Peripheral blood mononuclear cells*. After collection of blood into tubes containing EDTA, samples were diluted at a 1:1 ratio with PBS and added to a SepMate tube containing human Pancoll. During centrifugation (1,200*g* for 10 min at 4 °C), PBMCs were isolated by density gradient. The isolated cells in plasma were transferred to a tube and centrifuged again at 300*g* for 10 min at 4 °C. The isolated PBMCs were either used freshly or cryopreserved in liquid nitrogen, using serum-free cryopreservation medium (CTL-Cryo ABC Media Kit, Immunospot).

For analysis, samples were thawed quickly, washed twice with 1% BSA/PBS (300*g* for 10 min at 4 °C) and labeled using the following procedure: 10 min at 4 °C with Fc-block (Miltenyi) at a 1:50 dilution in FACS buffer (PBS + 2% FBS), followed by the surface antibody mix. The mix comprised: Thermo Fisher anti-human CD45RO-FITC (1:40 dilution), anti-CCR7^−^APC (1:40 dilution), anti-CD3-AF700 (1:50 dilution), Fixable Viability Dye eFluor 780 (1:1,000 dilution), anti-CD4-Pacific Blue (1:25 dilution) and BioLegend: anti-CD8-PerCP (1:25 dilution), in a total volume of 100 µl FACS buffer and incubated for 30 min at 4 °C. Antigen-experienced CD4^+^ T cells consisting of CD3^+^CD4^+^CD8^−^ effector memory (CD45RO^+^CCR7^−^), effector (CD45RO^−^CCR7^−^) and central memory (CD45RO^+^CCR7^+^) cells were collected using a FACS Aria Fusion flow cytometer with FACSDiva (BD Biosciences). After, cells were washed in 0.04% BSA/PBS and approximately 16,500 cells per sample were loaded onto the 10x chip.

*Cerebrospinal fluid*. Human CSF samples (3–6 ml) were processed within 1 h of lumbar puncture. After centrifugation at 300*g* for 10 min, the cell pellet was incubated in a 2-ml tube with the following TotalSeq-C antibodies: anti-human CD4, anti-CD8A and mouse IgG1 isotype control (BioLegend, 0.5 µg of each). Then, we followed the Cell Surface Labelling Protocol from 10x Genomics, but with all centrifugations done at 300*g* for 10 min. All cells were loaded on the 10x chip, with a maximum target cell number of 10,000.

*10x library preparation and sequencing*. Further processing was done following the manufacturer’s protocol using the Chromium Next GEM Single Cell VDJ v1.1. For CSF samples, the Feature Barcoding technology for Cell Surface Protein steps was also performed. Libraries were sequenced on an Illumina NovaSeq 6000 S4 using the following read lengths: 150 bp, read 1; 8 bp, i7 index; 150 bp, read 2.

#### Bioinformatic analysis

*CRISPR screen analysis*. The Galaxy platform^59^ was used for data analysis. For raw fastq files, Je-Demultiplex-Illu (Galaxy Version 1.2.1) was used for de-multiplexing, followed by Cutadapt (Galaxy version 4.4+galaxy0) and Trimmomatic (Galaxy Version 0.39+galaxy0) to get the 20-bp sgRNA sequence. Counts were then obtained with MAGeCK^20^ (version 0.5.7.1^+^) count. Normalization across samples was conducted in R^60^ (version 4.0.0+) after a 50 raw count threshold using the geometric mean per sgRNA for normalization, and sgRNAs with fewer than 50 counts in more than two replicates of the same tissue were discarded altogether. The MAGeCK test was run without normalization or zero removal, and otherwise default parameters on Galaxy, using the information of control NT sgRNA for noise correction. All further data processing was done with R.

*Genome-wide screen analysis and validation screen design*. For plotting of the control values in genome-wide analysis results (Fig. 1b and Extended Data Figs. 1 and 2), four random control sgRNAs were sampled with replacement from the control sgRNA pool and their log_2_(fold change) averaged, for a total of 800 combinations of control ‘genes’ with four different sgRNAs each, to model the variability observed for a control ‘gene’ in the MAGeCK analysis.

The selection of candidates for the validation screen was performed based on the MAGeCK results of the genome-wide screen. All genes in meninges versus blood and parenchyma versus blood comparisons with an absolute ‘neg|lfc’ or ‘pos|lfc’ > 0.5 were included. Genes from other comparisons (meninges or parenchyma versus spleen and spleen versus blood) were included when the absolute ‘neg|lfc’ or ‘pos|lfc’ > 1 and the number of ‘neg|goodsgrna’ ≥ 2 for negative ‘neg|lfc’ candidates or of ‘pos|goodsgrna’ ≥ 2 for positive ‘pos|lfc’ candidates, or the absolute ‘neg|lfc’ or ‘pos|lfc’ > 0.6 and the number of ‘neg|goodsgrna’ or ‘pos|goodsgrna’ > 2. Only genes expressed in T cells (based on ref. ^7^) were included for validation, except for genes from meninges or parenchyma versus blood if they had an absolute ‘neg|lfc’ or ‘pos|lfc’ > 0.85. In addition to the genes selected based on these criteria, we also included genes expressed in T cells (based on ref. ^7^) belonging to the GO terms GO:0004896 (cytokine receptor activity), GO:0050840 (extracellular matrix binding), GO:0004930 (GPCR activity), GO:0005178 (integrin binding) and GO:0033627 (cell adhesion mediated by integrin). We further included genes from selected gene sets from running GSEA (Broad) with default parameters except for ‘metric for ranking genes’ = ‘log2_Ratio_of_Classes’, databases of the GSEA molecular signature databases v7.0, and maximum and minimum sizes for excluding gene sets 20,000 and 5, respectively. The selected gene sets were GO:0043112 (receptor metabolic process), GO:0072583 (clathrin-dependent endocytosis), GO:0097384 (cellular lipid biosynthetic process), GO:0042092 (type 2 immune response), GO:0051955 (regulation of amino acid transport), GO:0098661 (inorganic anion transmembrane transport), GO:0015698 (inorganic anion transport), GO:0035655 (IL-18-mediated signaling pathway), GO:0071277 (cellular response to calcium ion), GO:0016574 (histone ubiquitination), GO:0043968 (histone H2A acetylation), GO:0006089 (lactate metabolic process), GO:0070670 (response to IL-4), GO:0032606 (type I interferon production) and GO:0002755 (MyD88-dependent toll-like receptor signaling pathway). For some GO terms, only the genes having a negative log_2_(fold change) when the GO term was negatively enriched or positive log_2_(fold change) when the GO term was positively enriched were included. This resulted in a total or 1,374 genes that were selected based on log2(fold change) and significance cutoffs in different comparisons and 587 genes that were selected based on GO terms, for a total of 1,961 genes (Supplementary Table 3).

*Identification of essential regulators of migration*. Validation screen candidates were considered essential regulators of T cell migration to the CNS when they met the following criteria as per the results of the MAGeCK analysis: ‘neg|lfc’ < −3 times the standard deviation of control (NT) ‘neg|lfc’ and ‘pos|lfc’ in the sample, the number of ‘neg|goodsgrna’ ≥ 3, and the ‘neg|p-value’ < 0.05; or ‘pos|lfc’ > 3 times the standard deviation of control ‘neg|lfc’ and ‘pos|lfc’ in the sample, the number of ‘pos|goodsgrna’ ≥ 3 and the ‘pos|p-value’ < 0.05.

*Bulk RNA-sequencing data*. Galaxy and R were also used for bulk RNA-seq data processing. Fastq files were aligned with RNA STAR (version 2.7.2b) to the reference genome Rnor_6.0.102 with default parameters and without trimming. HTSeq-count (version 1.0.0) was then used, and differential expression determined with DESeq2 (version 2.11.40.7+galaxy1), with ‘estimateSizeFactors’ = poscounts and otherwise default parameters. Batch correction per animal was performed when applicable. All further analysis was run with R, tidyverse^60,61^ (version 4.0.0+). For pathway analysis, the gProfiler^62^ web tool was used on genes with an adjusted *P* value < 0.05 in an ordered query from most to least extreme log_2_(fold change).

*Processing of single-cell sequencing data*. Sequencing results were de-multiplexed and aligned to the human GRCh38 reference genome using Cell Ranger (10X Genomics, v.6.1). Gene barcodes with unique molecular identifier counts that reached the threshold for cell detection were included in subsequent data analysis, using the R package Seurat^63^ (version 4.1.0).

*Analysis of human scRNA-seq data within compartments*. In a first step, data from the different compartments were analyzed separately. Data from cells with between 200 and 5,000 genes per cell and a percentage of mitochondrial genes below 10% were included in further analyses. Genes expressed in fewer than three cells were excluded. Data were log normalized and a batch effect was found in both the CSF and blood compartments. Therefore, established integration methods implemented within the Seurat package (CCA (canonical correlation analysis) and rPCA (reciprocal principal component analysis)) were applied, using the vst algorithm for detection of variable features. Data were then scaled and principal components computed for dimensional reduction. Applying *k*-nearest neighbors, the neighborhood overlap between cells was computed, followed by clustering the cells with a shared nearest-neighbor-based clustering algorithm. Uniform manifold approximation and projection was applied to visualize data in a two-dimensional space. The information from TCR enrichment library sequencing (10x Genomics, v.6.1) was added for each cell, followed by subsetting for cells where a TCR clonotype and only one beta chain had been detected. Small cell clusters with low quality and doublets, as well as a cluster expressing natural killer T cell signatures, were removed. For selecting the CD4^+^ T cells of the CSF for further analysis, the information provided by the anti-CD4 staining was used.

*Computing the TCR overlap between compartments*. For each individual MS or control sample, the amino acid sequence information from the TCR enrichment sequencing was used to match cells with identical TCR expression present in both the blood and CSF compartment of the same participant. If the expression of a TCR was found across compartments, cells were considered as overlapping. For TCRs found more or equal three times in total, cells were labeled as expanded across tissues.

*Combined analysis of human scRNA-seq data*. Both datasets (CSF and blood) were merged, log normalized, integrated with rPCA and scaled, before undergoing principal component analysis, neighborhood computation, clustering and dimensionality reduction using uniform manifold approximation and projection. The remaining 13 clusters were assigned a number, based on their expression level of CCR7 from high to low, with a Treg cluster characterized by Foxp3 expression. Each of the clusters expressed specific features, making them distinct on a transcriptomic level (Extended Data Fig. 8). Cluster T12 included only 13 cells from the CSF and was manually assigned to the nearest cluster T7 as it appeared to be unique for the blood compartment.

*Pathway analysis of human scRNA-seq data*. For analysis of enriched pathways in overlapping T cells compared to nonoverlapping cells derived from the blood of participants with MS (Extended Data Fig. 9), differentially expressed genes were computed using the R package MAST90 within the FindMarkers function of Seurat, with ‘logfc.threshold’ = 0, per cluster and overall. Genes enriched in overlapping versus nonoverlapping cells with an ‘avg_log2FC’ > two times the standard deviation of the ‘avg_log2FC’ of the individual comparison and a ‘p_val_adj’ < 0.05 were subset and the package EnrichR^64–66^ was used for pathway analysis using the database ‘GO_Biological_Process_2021’. Pathways with an ‘Adjusted.P.value’ < 0.05 and ‘Count’ ≥ 2 were considered significant and included in further analysis. For Extended Data Fig. 9c, the pathways plotted are derived from the comparison of all overlapping versus nonoverlapping cells (not taking into account the clusters), but only pathways that were enriched in overlapping cells compared to nonoverlapping cells in the overall comparison as well as in at least five other individual cluster-specific comparisons were included in the plot.

#### Schematic representation of functional modules

For Figs. 2i, 3g and 4j, the schematic representations of the essential modules regulating T cell migration were composed based on published studies, for the adhesion module^27,67–84^, for the chemotaxis module^22,30,85–88^ and for the egress module^33,89–93^. Color coding corresponds to parenchyma versus blood log_2_(fold change) values derived from the validation screen for those genes included in this screen (Supplementary Table 2); if the gene was not included due to not passing the initial selection criteria, color coding corresponds to the parenchyma versus blood log_2_(fold change) values derived from the genome-wide screen (Supplementary Table 1). For genes with more than one isoform, only those deemed relevant based on the literature are shown. When there were no published reports, only the isoforms included in the validation screen and thus showing a more pronounced log_2_(fold change) in the genome-wide screen were included. All parenchyma versus blood values used to generate the figures can be found in Supplementary Table 4.

*Statistical analysis and software*. FlowJo (version 10+) was used for analysis of flow cytometry data. For statistical analyses and plotting, GraphPad Prism version 7+ (GraphPad Software) and R^60,61^ (version 4.0.0+) were used. Calculations for total cell numbers were performed using R and Excel (Microsoft Office). Data are represented as the mean ± s.d. In box-and-whisker plots (Figs. 2, 3 and 4), the line in the middle of the box is the median, the box extends from 25th to 75th percentiles, whiskers extend from minimum to maximum values and the line connecting the box-and-whisker plots represents the mean Sample sizes are reported in the figure legends. All replicates are biological. Measurements were not repeated except in clinical-course experiments, where the same animal was tracked across several days. For disease-course experiments, the control animals were selected based on the same date of the cell transfer as for the KO-transferred animals. Some control animals in the disease-course analysis were therefore used for more than one KO. KO/control comparisons are represented as a ratio KO phenotype/control phenotype, unless otherwise specified. For the *Grk2*-KO/*S1pr1*-KO and double-KO experiment, the *Grk2*-KO values are the same as depicted previously for the *Grk2*-KO migration phenotype data (Fig. 4b,i). For in vitro surface staining experiments and intracellular cytokine stainings, control values are reused and therefore identical across different figures (Extended Data Figs. 4–7) as specified in the legends. The Shapiro–Wilk normality test was used to determine Gaussian distributions. When the dataset showed a Gaussian distribution, parametric tests were applied; in cases of a non-Gaussian distribution, non-parametric tests were used. The statistical analysis of CRISPR screen results was performed solely with the MAGeCK software^20^ using default settings (Figs. 1, 2, 3 and 4, Extended Data Figs. 1, 2 and 3 and Supplementary Tables 1, 2 and 4) and RNA-seq statistical analysis was performed solely by DESeq2 (galaxy; Fig. 6 and Extended Data Figs. 6 and 7), as described above. Test statistics were corrected for multiple testing if more than one comparison was run in parallel, with a two-stage linear step-up procedure of Benjamini, Krieger and Yekutieli. For the evaluation of KO T_MBP_ cell migration validation experiments by FACS, animals with <100 cells detected in any population were excluded. All KO/control phenotype datasets were statistically evaluated with one-sample *t*-tests (parametric) or Wilcoxon signed-rank tests (non-parametric). For the *Grk2*/*S1pr1*-KO experiment, an ordinary one-way ANOVA with Tukey’s multiple-comparison tests (parametric) and Kruskal–Wallis test with Dunn’s multiple-comparison test (non-parametric) were run for the KO/control phenotypes across the different KOs. For the assessment of the disease induction and weight change phenotypes of the KO cell transfer, a repeated-measures two-way ANOVA for time and genotype variations (days 3 to 8 after T_MBP_ cell transfer for disease score, and days 0 to 8 for weight changes) and Sidak’s multiple-comparison test were run. For surface and intracellular FACS staining experiments, two-way ANOVAs were run with multiple comparisons between control and KO cells, correcting for multiple comparisons with the two-stage set-up method of Benjamini, Krieger and Yekutieli for controlling for the false discovery rate. The statistical tests are reported in the figure legends, as well as two-way ANOVA *F* and *P* values corresponding to T_MBP_ cell genotype variation (one degree of freedom), KO T_MBP_ cells compared to control T_MBP_ cells. All other *F* and *P* values are not reported. All statistical tests and *P* values are two tailed. For the human data, differential expression of genes between MS and control conditions and blood and CSF were computed using the R package MAST90 within the FindMarkers function of Seurat (Fig. 7 and Extended Data Figs. 9 and 10). Correlations are Pearson correlations, as indicated in the figure legends. Both CRISPR screens have three replicates. One blood replicate from the genome-wide screen was excluded due to bad technical quality. The bulk RNA-seq for the *Ets1*-KO and *Grk2*-KO experiments have three replicates. All other *n* values are reported in the figure legends. Significance was set as: NS, *P* > 0.05; *P < 0.05, **P < 0.01, ***P < 0.001 and ****P < 0.0001. Adobe Illustrator (Adobe Systems), Inkscape and PowerPoint (Microsoft Office) were used for figure preparation.

#### Sample size, randomization and blinding

For the CRISPR screens, the sample size was determined based on the number of cells needed to maintain a minimum of 100× and a maximum of 1,000× coverage in all relevant cell populations, with the aim to minimize false discovery rates and ensure statistically meaningful data while also considering the practicality of handling the required number of cells. The number of replicates was chosen based on ref. ^94^.

No statistical methods were used to predetermine sample sizes, but our sample sizes are similar to those reported in previous publications (refs. ^5–7,18^). For the analysis of human CD4^+^ T cells by NGS, the samples from MS and control participants were selected due to availability of sufficient biomaterials.

The animals were allocated randomly for experimental groups. Data collection and analysis were not performed blind to the conditions of the experiments.

### Reporting summary

Further information on research design is available in the Nature Portfolio Reporting Summary linked to this article.

## Online content

Any methods, additional references, Nature Portfolio reporting summaries, source data, extended data, supplementary information, acknowledgements, peer review information; details of author contributions and competing interests; and statements of data and code availability are available at 10.1038/s41593-023-01432-2.

### Supplementary information

Reporting Summary

Supplementary Video 1 Time-lapse movie of control (lilac) and *Grk2*-KO TMBP cells (green) at the spinal cord leptomeninges at before onset of clinical EAE on day 2 after TMBP cell transfer. Blood vessels (gray) were visualized by intravenous injection of fluorescent dextran. Open arrow heads indicate the extravasated control and *Grk2*-KO TMBP cells. Inserted number indicates the time after the start of imaging. Scale bar, 50 μm.

Supplementary Video 2 Time-lapse movie of control (lilac) and *Grk2*-KO TMBP cells (green) at the spinal cord leptomeninges after onset of clinical EAE on day 3 after TMBP cell transfer. Blood vessels (gray) were visualized by intravenous injection of fluorescent dextran. White dashed lines outline blood vessels. Inserted number indicates the time after the start of imaging. Scale bar, 50 μm.

Supplementary Table 1 Genome-wide screen Mageck results.

Supplementary Table 2 Validation screen Mageck results.

Supplementary Table 3 Primers, oligonucleotides and PCR protocols.

Supplementary Table 4 Module cartoon schemes raw data.

Supplementary Table 5 Materials company names and catalog numbers.

Supplementary Code 1 All analysis code not for human scRNA-seq.

Supplementary Code 2 Analysis of CD4^+^ human T cells scRNA-seq—combination of PBMCs and CSF cells.

Supplementary Code 3 Analysis of CD4^+^ human T cells scRNA-seq—fold change of interesting genes MS versus control.

Supplementary Code 4 Analysis of CD4^+^ human T cells scRNA-seq—figure making.

Supplementary Code 5 Analysis of CD4^+^ human T cells scRNA-seq—calculating overlapping cells.

### Source data

Source Data Fig. 1 Figure and statistical source data.

Source Data Fig. 2 Figure and statistical source data.

Source Data Fig. 3 Figure and statistical source data.

Source Data Fig. 4 Figure and statistical source data.

Source Data Fig. 5 Figure and statistical source data.

Source Data Fig. 5 Unprocessed western blot images and information file about how to open them.

Source Data Fig. 6 Figure and statistical source data.

Source Data Extended Data Fig. 1 Figure and statistical source data.

Source Data Extended Data Fig. 2 Figure and statistical source data.

Source Data Extended Data Fig. 3 Figure and statistical source data.

Source Data Extended Data Fig. 4 Figure and statistical source data.

Source Data Extended Data Fig. 5 Figure and statistical source data.

Source Data Extended Data Fig. 6 Figure and statistical source data.

Source Data Extended Data Fig. 7 Figure and statistical source data.

Source Data Extended Data Fig. 7 Unprocessed western blot images and information file about how to open them.

Source Data Extended Data Fig. 9 Figure and statistical source data.

## Data Availability

NGS raw data and processed gene expression data that support the findings of this study are deposited in the Gene Expression Omnibus under the accession number GSE232344 (GSE232340 for data on the CRISPR screens, GSE232339 for bulk RNA-seq data and GSE232343 for scRNA-seq data). Source data are provided with this paper. All other data generated or analyzed during this study are included in the published article or are available from the corresponding authors upon reasonable request.

## Code availability

Custom code used for the analysis of the data for this paper is available as Supplementary Code 1–5.
