# Supplementary material for: A genome-wide in vivo CRISPR screen identifies essential regulators of T cell migration to the CNS in a multiple sclerosis model
Source: Nat Neurosci. 2023 Sep 14;26(10):1713–25. doi: 10.1038/s41593-023-01432-2 (PMC10545543; doi:10.1038/s41593-023-01432-2)
Supplement: Supplementary file 1 — Reporting Summary [file 41593_2023_1432_MOESM1_ESM.pdf]

nature portfolio

Corresponding author(s): Martin Kerschensteiner
Naoto Kawakami

Last updated by author(s): Aug 4, 2023

Reporting Summary

Nature Portfolio wishes to improve the reproducibility of the work that we publish. This form provides structure for consistency and transparency in reporting. For further information on Nature Portfolio policies, see our Editorial Policies and the Editorial Policy Checklist.

Statistics

For all statistical analyses, confirm that the following items are present in the figure legend, table legend, main text, or Methods section.

n/a	Confirmed	
☐	☒	The exact sample size (*n*) for each experimental group/condition, given as a discrete number and unit of measurement
☐	☒	A statement on whether measurements were taken from distinct samples or whether the same sample was measured repeatedly
☐	☒	The statistical test(s) used AND whether they are one- or two-sided<br>*Only common tests should be described solely by name; describe more complex techniques in the Methods section.*
☐	☒	A description of all covariates tested
☐	☒	A description of any assumptions or corrections, such as tests of normality and adjustment for multiple comparisons
☐	☒	A full description of the statistical parameters including central tendency (e.g. means) or other basic estimates (e.g. regression coefficient) AND variation (e.g. standard deviation) or associated estimates of uncertainty (e.g. confidence intervals)
☐	☒	For null hypothesis testing, the test statistic (e.g. *F*, *t*, *r*) with confidence intervals, effect sizes, degrees of freedom and *P* value noted<br>*Give P values as exact values whenever suitable.*
☒	☐	For Bayesian analysis, information on the choice of priors and Markov chain Monte Carlo settings
☒	☐	For hierarchical and complex designs, identification of the appropriate level for tests and full reporting of outcomes
☐	☒	Estimates of effect sizes (e.g. Cohen's *d*, Pearson's *r*), indicating how they were calculated

Our web collection on statistics for biologists contains articles on many of the points above.

Software and code

Policy information about availability of computer code

Data collection	Galaxy platform (Je-Demultiplex-Illu (Galaxy Version 1.2.1), Cutadapt (Galaxy Version 4.4+galaxy0), Trimmomatic (Galaxy Version 0.39 +galaxy0)), FACS Suite 1.06 (FACS VERSE), FACSDiva 8.0 (BD FACS Aria Fusion, FACS Fortessa), CytExpert 2.5.0.77 (Cytoflex S), Image Studio Lite Software version 5.2 (Odyssey FC, OFC-0661), Bio-Rad CFX Maestro 1.1 version 4.1.2433.1219 (Bio-Rad CFX Connect Real-Time PCR system), ICE software tool (version 2.0+)
Data analysis	MAGeCK (version 0.5.7.1+), R (version 4.0.0+), tidyverse, Seurat v.4.1.0, MAST v.1.16.0, enrichR (v3.1), Cell ranger 10X Genomics v.6.1, Fiji (version 1.53e), RNA STAR (version 2.7.2b), HTSeq-count (version 1.0.0), DESeq2 (version 2.11.40.7+galaxy1), gProfiler (version of 2019), FlowJO (version 10+), GraphPad Prism (version 7+), Excel (Microsoft Office, 2016+)<br>Custom code used for the analysis of the data for this paper are uploaded as supplementary code in the supplementary information.

For manuscripts utilizing custom algorithms or software that are central to the research but not yet described in published literature, software must be made available to editors and reviewers. We strongly encourage code deposition in a community repository (e.g. GitHub). See the Nature Portfolio guidelines for submitting code & software for further information.

Data

Policy information about availability of data

All manuscripts must include a data availability statement. This statement should provide the following information, where applicable:

- Accession codes, unique identifiers, or web links for publicly available datasets
- A description of any restrictions on data availability
- For clinical datasets or third party data, please ensure that the statement adheres to our policy

Next-generation sequencing raw data and processed gene expression data that support the findings of this study are deposited into GEO under the accession

number GSE232344 (GSE232340 for data on the CRISPR screens, GSE232339 for bulk RNAseq data and GSE232343 for scRNAseq data). All other data generated or analysed during this study is included in the published article or is available from the corresponding authors upon reasonable request.

Field-specific reporting

Please select the one below that is the best fit for your research. If you are not sure, read the appropriate sections before making your selection.

☒ Life sciences ☐ Behavioural & social sciences ☐ Ecological, evolutionary & environmental sciences

For a reference copy of the document with all sections, see nature.com/documents/nr-reporting-summary-flat.pdf

Life sciences study design

All studies must disclose on these points even when the disclosure is negative.

Sample size	For the CRISPR screens, the sample size was determined based on the number of cells needed to maintain a minimum of 100x and a maximum of 1,000x coverage in all relevant cell populations, with the aim to minimize false discovery rates and ensure statistically meaningful data, while also considering the practicality of handling the required number of cells. Replicate number was chosen based on Ong et al, Scientific Reports 2017.<br>No statistical methods were used to pre-determine sample sizes but our sample sizes are similar to those reported in previous publications (See Ref. Odoardi et al., Nature 2012, Bartholomäus et al., Nature 2009, Schläger et al., Nature 2016, Kawakami et al., Journal of Experimental Medicine 2004). For the analysis of human CD4+ T cells by NGS, the samples from MS and control patients were selected due to availability of sufficient biomaterials.
Data exclusions	For in vivo migration assay (co-transfer experiments, Fig. 2c,g; 3b; 4b,i; 6b; Extended Data Fig. 5e,g), FACS data samples from the whole animal were excluded when <100 individual cells were detected in any population of interest.
Replication	All animal experiments in this study include at least three independent biological replicates. The number of replicates is mentioned in the corresponding figure legend of each experiment. All replicated experiments were successful, except when prior exclusion criteria were met.<br><br>15,768 and 807 CSF derived and 29,749 and 40,845 blood derived CD4+ T cells from 4 MS patients and 4 control patients were used for NGS, respectively
Randomization	Rats were randomly allocated into experimental groups.<br><br>Human samples were allocated by MS patients and control patients. The samples were not randomized.
Blinding	Investigators were not blinded during experiments due to the nature of the binary type of experiments (e.g. co-transfer experiments: BFP vs GFP labeled cell, disease course: obvious disease phenotype control vs EAE).<br><br>The analysis of NGS data is unbiased. Therefore, investigators were not blinded.

Reporting for specific materials, systems and methods

We require information from authors about some types of materials, experimental systems and methods used in many studies. Here, indicate whether each material, system or method listed is relevant to your study. If you are not sure if a list item applies to your research, read the appropriate section before selecting a response.

Materials & experimental systems

n/a	Involved in the study	
☐	☒	Antibodies
☐	☒	Eukaryotic cell lines
☒	☐	Palaeontology and archaeology
☐	☒	Animals and other organisms
☐	☒	Human research participants
☒	☐	Clinical data
☒	☐	Dual use research of concern

Methods

n/a	Involved in the study	
☒	☐	ChIP-seq
☐	☒	Flow cytometry
☒	☐	MRI-based neuroimaging

Antibodies

Antibodies used	unconjugated mouse IgG1 Isotype control (Sigma, Cat#: M-1398, clone MOPC31c, Lot#: 084K4862, dilution: 1:100),<br>unconjugated NA/LE armenian hamster IgG2 Isotype control (BD Pharmingen, Cat#: 553961, clone Ha4/8, Lot#: 5105640, dilution: 1:100),<br>rat IgG1 Isotype control-PE (BD Pharmingen, Cat#: 554685, clone R3-34, Lot#: 87970, dilution: 1:100),<br>unconjugated mouse anti-rat CD49d (Thermo Fisher, Cat#: MA49D7, clone TA-2, Lot#: WG334936, dilution: 1:100),<br>unconjugated mouse anti-rat CD11a (Biolegend, Cat#: 201902, clone wt.1, Lot#: B267211, dilution: 1:100),

unconjugated mouse anti-rat CD18 (Thermo Fisher, Cat#: MA1817, clone wt.3, Lot#: RK245842, dilution: 1:100),
unconjugated mouse anti-rat TCRbeta (BD Pharmingen, Cat#: 554911, clone R73, Lot#: 0199116, dilution: 1:100),
unconjugated mouse anti-rat CD25 (Thermo Fisher, Cat#: MA517490, clone OX39, Lot#: VJ3104933, dilution: 1:100),
unconjugated mouse anti-rat CD134 (Thermo Fisher, Cat#: MA1-70020, clone OX40, Lot#: YC3847111, dilution: 1:100),
unconjugated armenian hamster anti-rat CD29 (Biolegend, Cat#: 102202, clone HMβ1-1, Lot#: B326869, dilution: 1:100),
rat anti-mouse/rat IL-17A-PE (BD Pharmingen, Cat#: 559502, clone TC11-18H10, Lot#: 81990, dilution: 1:100),
unconjugated mouse anti-rat IFNγ (eBioscience, Cat#: 14-7310-85, clone DB1, Lot#: E05481-500, dilution: 1:100),
donkey anti-mouse IgG-APC (Jackson ImmunoResearch, Cat#: 715-136-151, polyclonal, Lot#: 122609, dilution: 1:1000),
goat anti-armenian hamster IgG-APC (Jackson ImmunoResearch, Cat#: 127-135-160, polyclonal, Lot#: 150828, dilution: 1:1000),
goat anti-mouse IgG-AF647 (SouthernBiotech, Cat#1038-31, polyclonal, Lot#: C1717-NI90C, dilution: 1:1000),
mouse anti-human-CD4-FITC (Biolegend, Cat#: 344604, clone SK3, Lot#: B331829, dilution: 1:100),
mouse anti-human-S1PR1-eF660 (Thermo Fisher, Cat#: 50-3639-41, clone SW4GYPP, Lot#: 2349796, dilution: 1:100),
mouse anti-human CD45RO-FITC (Thermo Fisher, Cat#: 11-0457-42, clone UCHL1, Lot#: 4272459, dilution: 1:40),
rat anti-human CCR7-APC (Thermo Fisher, Cat#: 17-1979-42, clone 3D12, Lot#: 4290631, dilution: 1:40),
mouse anti-human CD3-AF700 (Thermo Fisher, Cat#: 56-0037-42, clone OKT3, Lot#: 4330031, dilution: 1:50),
mouse anti-human CD4-Pacific Blue (Thermo Fisher, Cat#: MHCD0428, clone S3.5, Lot#: 2123513, dilution: 1:25),
mouse anti-human CD8-PerCP (BioLegend, Cat#: 344708, clone SK1, Lot#: B204989, dilution: 1:25),
rabbit anti-rat ETS1 (Cell Signaling Technology, Cat#: 14069S, clone D808A, Lot #: 3, dilution: 1:1000),
mouse anti-beta Actin Antibody HRP (Santa Cruz, Cat#: sc-47778 HRP, clone C4, Lot #: A2418, dilution: 1:100000),
mouse anti-rabbit IgG-HRP (Santa Cruz, Cat#: sc-2357 HRP, secondary antibody, Lot #: A0318, dilution: 1:10000),
rabbit anti-GRK2 (Cell Signaling Technology, Cat#: 74761S, polyclonal, Lot #: 1, dilution: 1:1000),
rabbit anti-Phospho-p44/42 MAPK(Erk1/2)(Thr202/Tyr204) (Cell Signaling Technology, Cat#: 4370T, clone D13.14.4E, Lot #: 28, dilution: 1:1000),

rabbit anti-p44/42 MAPK(Erk1/2) (Cell Signaling Technology, Cat#: 4695T, clone 137F5, Lot #: 35, dilution: 1:1000),
anti-human-CD49d-APC (Biolegend, Cat#: 304307, clone 9F10, Lot#: B270194, dilution: 1:100),
anti-human CXCR3-PE (Biolegend, Cat#: 353705, clone G025H7, Lot#: B342049, dilution: 1:100),
anti-human CD29-PE (Biolegend, Cat#: 303003, clone TS2/16, Lot#: B358184, dilution: 1:100),
anti-human CD11a-PE (Biolegend, Cat#: 301207, clone HI111, Lot#: B319403, dilution: 1:100),
mouse anti-human CD3 (Thermo Fisher, Cat#: 16-0037-81, clone OKT3, Lot#: 2493171),
mouse anti-human CD28 (Thermo Fisher, Cat#: 16-0289-81, clone CD28.2, Lot#: 2470259)
anti-human CD4 (Catalog no: 300567; Lot no: B300113; Clone: RPA-T4)
anti-human CD8a (Catalog no: 301071; Lot no: B315106; Clone: RPA-T8)
isotype mIgG1-control-Ab (Catalog no: 400187; Lot no: B333559; Clone: MOPC-21)

Validation

Antibodies were commercially available and validated by the manufacturers or used in previous studies according to manufactures:
Flow cytometry:
unconjugated mouse IgG1 Isotype control (Sigma, Cat#: M-1398) : 8 citations
unconjugated NA/LE armenian hamster IgG2 Isotype control (BD Pharmingen, Cat#: 553961) : 4 citations
rat IgG1 Isotype control-PE (BD Pharmingen, Cat#: 554685) : data sheet
unconjugated mouse anti-rat CD49d (Thermo Fisher, Cat#: MA49D7) : data sheet
unconjugated mouse anti-rat CD11a (Biolegend, Cat#: 201902) : data sheet
unconjugated mouse anti-rat CD18 (Thermo Fisher, Cat#: MA1817) : data sheet
unconjugated mouse anti-rat TCRbeta (BD Pharmingen, Cat#: 554911) : data sheet
unconjugated mouse anti-rat CD25 (ThermoFisher, Cat#: MA517490) : data sheet
unconjugated mouse anti-rat CD134 (Invitrogen, Cat#: MA1-70020) : data sheet
unconjugated armenian hamster anti-rat CD29 (Biolegend, Cat#: 102202) : data sheet
rat anti-mouse IL-17A-PE (BD Pharmingen, Cat#: 559502) : data sheet
unconjugated mouse anti-rat IFNγ (eBioscience, Cat#: 14-7310-85) : 38 citations
mouse anti-human-CD4-FITC (Biolegend, Cat#: 344604) : data sheet
mouse anti-human-S1PR1-eF660 (Thermo Fisher, Cat#: 50-3639-41) : data sheet
mouse anti-human CD45RO-FITC (Thermo Fisher, Cat#: 11-0457-42) : data sheet
rat anti-human CCR7-APC (Thermo Fisher, Cat#: 17-1979-42) : data sheet
mouse anti-human CD3-AF700 (Thermo Fisher, Cat#: 56-0037-42) : data sheet
mouse anti-human CD4-Pacific Blue (Thermo Fisher, Cat#: MHCD0428) : data sheet
mouse anti-human CD8-PerCP (BioLegend, Cat#: 344708) : data sheet
mouse anti-human-CD49d-APC (Biolegend, Cat#: 304307) : data sheet
mouse anti-human CXCR3-PE (Biolegend, Cat#: 353705) : data sheet
mouse anti-human CD29-PE (Biolegend, Cat#: 303003) : data sheet
mouse anti-human CD11a-PE (Biolegend, Cat#: 301207) : data sheet
Westernblot:
rabbit anti-rat ETS1 (Cell Signaling Technology, Cat#: 14069S) : data sheet
mouse anti-beta Actin Antibody HRP (Santa Cruz, Cat#: sc-47778 HRP) : data sheet
rabbit anti-GRK2 (Cell Signaling Technology, Cat#: 74761S) : data sheet
rabbit anti-Phospho-p44/42 MAPK(Erk1/2)(Thr202/Tyr204) (Cell Signaling Technology, Cat#: 4370T) : data sheet
rabbit anti-p44/42 MAPK(Erk1/2) (Cell Signaling Technology, Cat#: 4695T) : data sheet
Functional assay:
mouse anti-human CD3 (Thermo Fisher, Cat#: 16-0037-81): 95 citations
mouse anti-human CD28 (Thermo Fisher, Cat#: 16-0289-81): 32 citations
scRNAseq:
anti-human CD4 (Catalog no: 300567; Lot no: B300113; Clone: RPA-T4): 8 citations
anti-human CD8a (Catalog no: 301071; Lot no: B315106; Clone: RPA-T8): 2 citations
isotype mIgG1-control-Ab (Catalog no: 400187; Lot no: B333559; Clone: MOPC-21): 3 citations

Eukaryotic cell lines

Policy information about cell lines

Cell line source(s)	HEK293 T cells (ATCC)<br>GP+E86 (ATCC)
Authentication	Cell line was not authenticated after purchase
Mycoplasma contamination	Cell line was not tested for mycoplasma contamination but no indication of contamination was observed
Commonly misidentified lines<br>(See ICLAC register)	No commonly misidentified cell lines were used.

Animals and other organisms

Policy information about studies involving animals; ARRIVE guidelines recommended for reporting animal research

Laboratory animals	Lewis rats (LEW/Crl and LEW/OrlRj) were obtained from Charles River and Janvier. They were bred at the animal facility of Biomedical center, LMU. Both male and female rats at age between 5-20 weeks were used
Wild animals	The study did not involve wild animals.
Field-collected samples	The study did not involve samples collected from the field.
Ethics oversight	Regierung von Oberbayern

Note that full information on the approval of the study protocol must also be provided in the manuscript.

Human research participants

Policy information about studies involving human research participants

Population characteristics	Subjects in the MS group had been diagnosed with relapsing-remitting MS according to the revised McDonald criteria and had a relapse no longer than 45 days prior to lumbar puncture. All patients are female and age 27, 31, 32 and 41. Samples from four sex- and age (25, 25, 27 and 52)- matched individuals diagnosed with idiopathic intracranial hypertension were included in the control group. PBMCs of four healthy donors used for CRISPR gene editing were derived from leukoreduction system chambers.
Recruitment	Patients with MS recruited for this study were seen at our outpatient department of the Institute of Clinical Neuroimmunology for clinical diagnostic work-up and CSF sampling was performed due to diagnostic purposes only. To reduce heterogeneity as well as confounders based on disease-modifying treatment, the RRMS-CSF cohort is composed of treatment naïve patients with early MS only and only those patients were eligible for our study. Hence with regard to patient selection we can exclude any self-selection bias or other biases and the selection of patients has to the best of our knowledge no impact on the results. As non-inflammatory controls we obtained and analysed CSF samples from patients with idiopathic intracranial hypertension. In these cases lumbar puncture was performed as a therapeutic intervention only.<br><br>We hereby confirm that all MS patients and controls (patients with IIH) included in the study have given a written informed consent. Recruitment of individuals took place from August 2020 to January 2021
Ethics oversight	Written informed consent was obtained from all subjects according to the Declaration of Helsinki, Collection of samples was approved by local ethics committees of the LMU, Munich, ethical vote: 163-16 and LMU #18-821

Note that full information on the approval of the study protocol must also be provided in the manuscript.

Flow Cytometry

Plots

Confirm that:

- [x] The axis labels state the marker and fluorochrome used (e.g. CD4-FITC).
- [x] The axis scales are clearly visible. Include numbers along axes only for bottom left plot of group (a 'group' is an analysis of identical markers).
- [x] All plots are contour plots with outliers or pseudocolor plots.
- [x] A numerical value for number of cells or percentage (with statistics) is provided.

Methodology

Sample preparation	Blood was withdrawn by heart puncture into a heparinized syringe. Spleen, parathymic lymph nodes, and leptomeninges and parenchyma of the spinal cord were dissected and homogenized by passing through a metal strainer. Lymphocytes were

isolated from blood by a Nycoprep gradient. First, the blood was diluted with an equal volume of PBS and overlaid onto Nycoprep. After centrifugation at 800 g, room temperature for 30 min with mild acceleration and brake, lymphocytes were collected from the interface. For spleen, erythrocytes were removed by treating with ACK buffer for 3 min on ice and lymphocytes were isolated using the EasySep™ Rat CD4+ T Cell Isolation Kit (StemCell, 19642), before purification by sorting. From the spinal cord parenchyma the lymphocytes were isolated using a 30%/64% Percoll gradient and centrifugation at 1,200 g, room temperature for 30 min with mild acceleration and brake. Lymphocytes were collected from the interface. In vitro lymphocytes were seperated from thymocytes by overlaying onto Nycoprep. After centrifugation at 675 g, room temperature for 10 min with mild acceleration and brake, lymphocytes were collected from the interface.

Instrument

FACS VERSE (BD Biosciences), FACS Fortessa (BD Biosciences), Cytoflex S (Beckman Coulter)

Software

FACS Suite (FACS VERSE), FACSDiva (FACS Fortessa), CytExpert (Cytoflex S), FlowJO (version 10+)

Cell population abundance

Cell population abundance pre-sorting ranged from 1% to >50% depending on the tissue. Post sort, purity was confirmed by re-analysis of the sorted population.

Gating strategy

Sorting rat T cells in genome-wide and validation screening: Lymphocytes were gated in an FSC-A vs. SSC-A dot plot defined by size and granularity. Doublets were excluded in a FSC-H vs FSC-A plot. Cells in the gate were displayed in a FITC-A vs. PB450-A/BV421-A plot to identify BFP+ and GFP+ populations.

In vivo migration analysis, chemotaxis assay: Lymphocytes were gated in an FSC-A vs. SSC-A dot plot defined by size and granularity. Cells in the gate were displayed in a FITC-A (EGFP) vs. PB450-A/BV421-A (BFP) plot to identify BFP+ and GFP+ populations.

In vitro rat T cell staining: Lymphocytes were gated in an FSC-A vs. SSC-A dot plot defined by size and granularity. Doublets were excluded in a FSC-H vs FSC-A plot. Cells in the gate were displayed in a FITC-A (EGFP) vs. PB450-A/BV421-A (BFP) plot to identify BFP+ and EGFP+ populations. The populations were further displayed in a single-parameter histogram of APC-A (CD49d/CD29/CD11a/CD18/TCR/CD25/CD134)

In vitro human T cell staining: Lymphocytes were gated in an FSC vs. SSC dot plot defined by size and granularity. Doublets were excluded first in a FSC-H vs FSC-A plot second in a SSC-H vs SSC-A plot. Cells in the gate were displayed in a FITC-A (CD4) vs. PB450-A (LIVE/DEAD) plot to identify live CD4+ T cells. The populations were further displayed in a single-parameter histogram of APC-A/PE (S1PR1, CD49d / CXCR3, CD11a, CD18).

Sorting human T cells: CD3+ T cell lymphocytes were gated in a SSC vs. AF700 (CD3) plot. The gated population was further separated into CD8+ and CD4+ populations in a PerCP (CD8) vs. PacificBlue (CD4) plot. Cells in each gate were displayed in a FITC (CD45RO) vs. APC (CCR7) plot to distinguish Effector Memory cells (CD45RO+, CCR7-), Central Memory Cells (CD45RO+, CCR7+), Effector cells (CD45RO-, CCR7-) and naïve cells (CD45RO-, CCR7+).

☒ Tick this box to confirm that a figure exemplifying the gating strategy is provided in the Supplementary Information.
