# Supplementary material for: A genome-wide in vivo CRISPR screen identifies essential regulators of T cell migration to the CNS in a multiple sclerosis model
Source: Nat Neurosci. 2023 Sep 14;26(10):1713–25. doi: 10.1038/s41593-023-01432-2 (PMC10545543; doi:10.1038/s41593-023-01432-2)
Supplement: Supplementary file 19 — Unprocessed western blot images and information file about how to open them. [file 41593_2023_1432_MOESM19_ESM.zip › F5D_WB_SourceData/for rep image 2023.03.12 HumanT cells NT and Grk2 UnS_10min/hTcells_Grk2_20230315_pERK/Grk2KO_pERK_tERK_representative figure.pptx]

## Slide 1
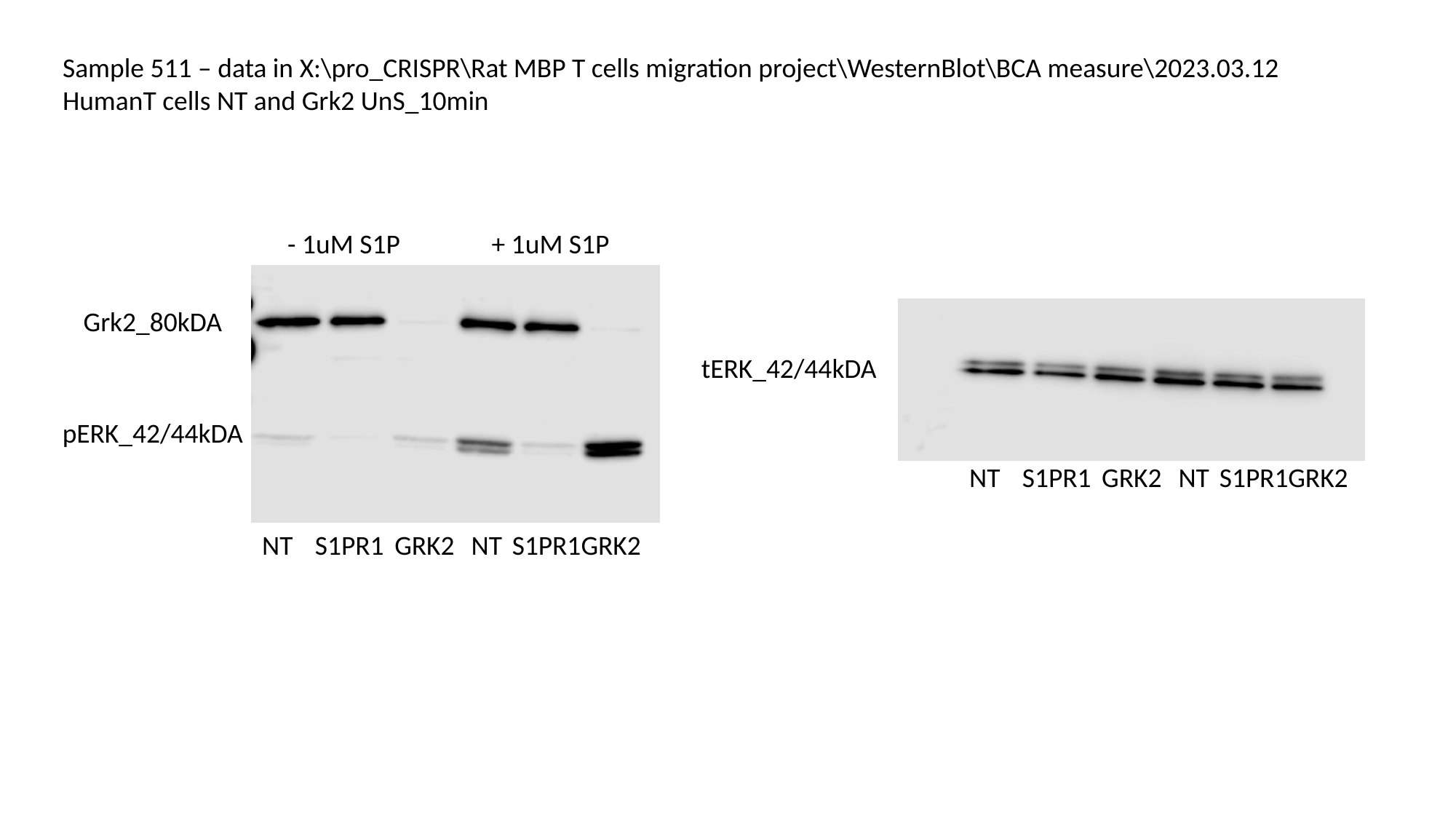

Sample 511 – data in X:\pro_CRISPR\Rat MBP T cells migration project\WesternBlot\BCA measure\2023.03.12 HumanT cells NT and Grk2 UnS_10min
- 1uM S1P
+ 1uM S1P
Grk2_80kDA
pERK_42/44kDA
NT
S1PR1
GRK2
NT
S1PR1
GRK2
tERK_42/44kDA
NT
S1PR1
GRK2
NT
S1PR1
GRK2
