# Supplementary figures and images for: A genome-wide in vivo CRISPR screen identifies essential regulators of T cell migration to the CNS in a multiple sclerosis model
Source: Nat Neurosci. 2023 Sep 14;26(10):1713–25. doi: 10.1038/s41593-023-01432-2 (PMC10545543; doi:10.1038/s41593-023-01432-2)

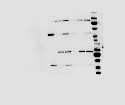

Supplement: Supplementary file 19 — Unprocessed western blot images and information file about how to open them. [file 41593_2023_1432_MOESM19_ESM.zip › F5D_WB_SourceData/for rep image 2023.03.12 HumanT cells NT and Grk2 UnS_10min/hTcells_Grk2_20230315_pERK/Images/0000771_01/0000771_01_TH.jpg]

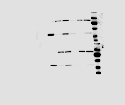

Supplement: Supplementary file 19 — Unprocessed western blot images and information file about how to open them. [file 41593_2023_1432_MOESM19_ESM.zip › F5D_WB_SourceData/for rep image 2023.03.12 HumanT cells NT and Grk2 UnS_10min/hTcells_Grk2_20230315_pERK/Images/0000772_01/0000772_01_TH.jpg]

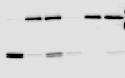

Supplement: Supplementary file 19 — Unprocessed western blot images and information file about how to open them. [file 41593_2023_1432_MOESM19_ESM.zip › F5D_WB_SourceData/for rep image 2023.03.12 HumanT cells NT and Grk2 UnS_10min/hTcells_Grk2_20230315_pERK/Images/0000772_02/0000772_02_TH.jpg]

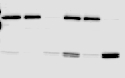

Supplement: Supplementary file 19 — Unprocessed western blot images and information file about how to open them. [file 41593_2023_1432_MOESM19_ESM.zip › F5D_WB_SourceData/for rep image 2023.03.12 HumanT cells NT and Grk2 UnS_10min/hTcells_Grk2_20230315_pERK/Images/0000772_03/0000772_03_TH.jpg]

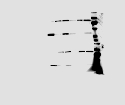

Supplement: Supplementary file 19 — Unprocessed western blot images and information file about how to open them. [file 41593_2023_1432_MOESM19_ESM.zip › F5D_WB_SourceData/for rep image 2023.03.12 HumanT cells NT and Grk2 UnS_10min/hTcells_Grk2_20230315_pERK/Images/0000773_01/0000773_01_TH.jpg]

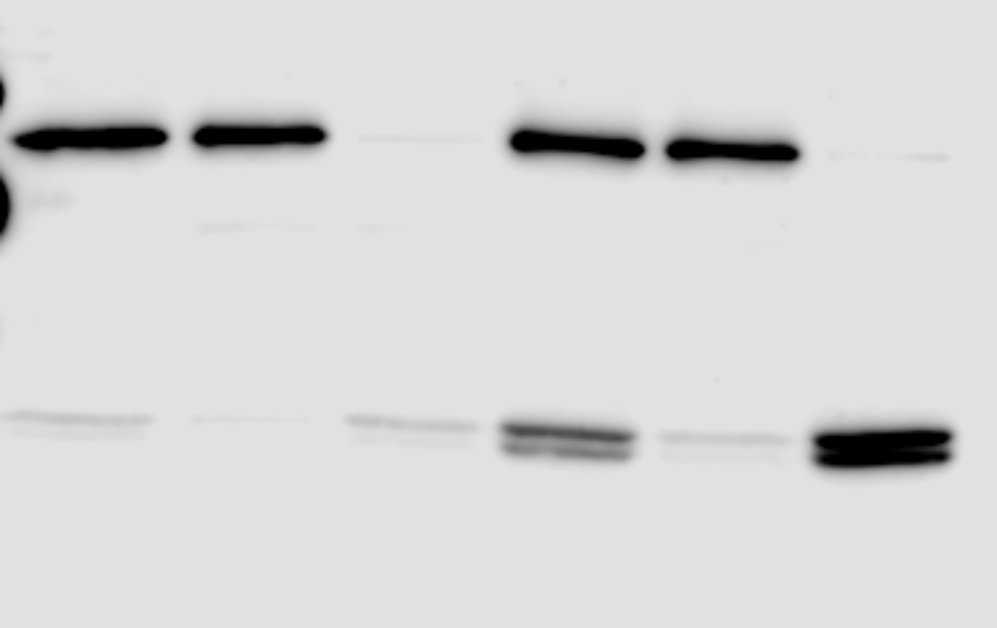

Supplement: Supplementary file 19 — Unprocessed western blot images and information file about how to open them. [file 41593_2023_1432_MOESM19_ESM.zip › F5D_WB_SourceData/for rep image 2023.03.12 HumanT cells NT and Grk2 UnS_10min/hTcells_Grk2_20230315_pERK/Rep_Fig1_pERK.png]

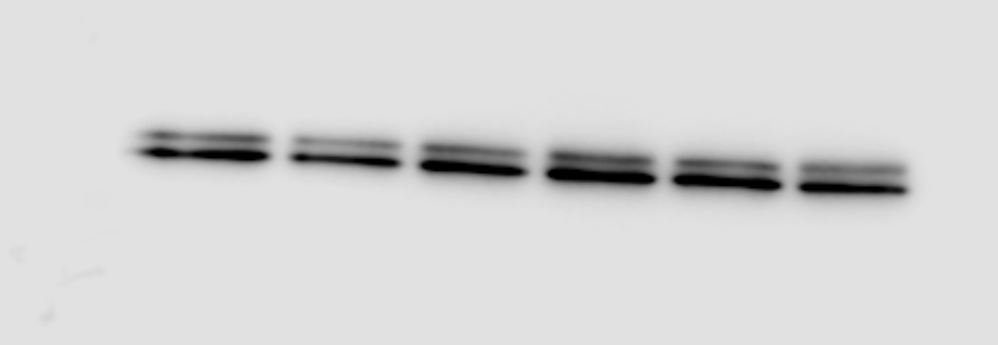

Supplement: Supplementary file 19 — Unprocessed western blot images and information file about how to open them. [file 41593_2023_1432_MOESM19_ESM.zip › F5D_WB_SourceData/for rep image 2023.03.12 HumanT cells NT and Grk2 UnS_10min/hTcells_Grk2_20230316_R2_tERK/518_repfig1_tERK.png]

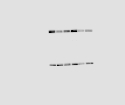

Supplement: Supplementary file 19 — Unprocessed western blot images and information file about how to open them. [file 41593_2023_1432_MOESM19_ESM.zip › F5D_WB_SourceData/for rep image 2023.03.12 HumanT cells NT and Grk2 UnS_10min/hTcells_Grk2_20230316_R2_tERK/Images/0000774_01/0000774_01_TH.jpg]

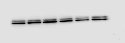

Supplement: Supplementary file 19 — Unprocessed western blot images and information file about how to open them. [file 41593_2023_1432_MOESM19_ESM.zip › F5D_WB_SourceData/for rep image 2023.03.12 HumanT cells NT and Grk2 UnS_10min/hTcells_Grk2_20230316_R2_tERK/Images/0000774_02/0000774_02_TH.jpg]

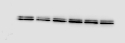

Supplement: Supplementary file 19 — Unprocessed western blot images and information file about how to open them. [file 41593_2023_1432_MOESM19_ESM.zip › F5D_WB_SourceData/for rep image 2023.03.12 HumanT cells NT and Grk2 UnS_10min/hTcells_Grk2_20230316_R2_tERK/Images/0000774_03/0000774_03_TH.jpg]

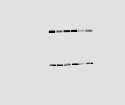

Supplement: Supplementary file 19 — Unprocessed western blot images and information file about how to open them. [file 41593_2023_1432_MOESM19_ESM.zip › F5D_WB_SourceData/for rep image 2023.03.12 HumanT cells NT and Grk2 UnS_10min/hTcells_Grk2_20230316_R2_tERK/Images/0000775_01/0000775_01_TH.jpg]

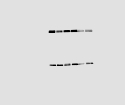

Supplement: Supplementary file 19 — Unprocessed western blot images and information file about how to open them. [file 41593_2023_1432_MOESM19_ESM.zip › F5D_WB_SourceData/for rep image 2023.03.12 HumanT cells NT and Grk2 UnS_10min/hTcells_Grk2_20230316_R2_tERK/Images/0000776_01/0000776_01_TH.jpg]

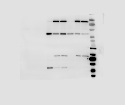

Supplement: Supplementary file 19 — Unprocessed western blot images and information file about how to open them. [file 41593_2023_1432_MOESM19_ESM.zip › F5D_WB_SourceData/hTcells_Grk2_20230228_pERK&Grk2_R1&R2/Images/0000758_01/0000758_01_TH.jpg]

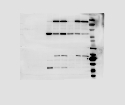

Supplement: Supplementary file 19 — Unprocessed western blot images and information file about how to open them. [file 41593_2023_1432_MOESM19_ESM.zip › F5D_WB_SourceData/hTcells_Grk2_20230228_pERK&Grk2_R1&R2/Images/0000759_01/0000759_01_TH.jpg]

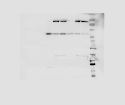

Supplement: Supplementary file 19 — Unprocessed western blot images and information file about how to open them. [file 41593_2023_1432_MOESM19_ESM.zip › F5D_WB_SourceData/hTcells_Grk2_20230228_pERK&Grk2_R1&R2/Images/0000760_01/0000760_01_TH.jpg]

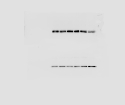

Supplement: Supplementary file 19 — Unprocessed western blot images and information file about how to open them. [file 41593_2023_1432_MOESM19_ESM.zip › F5D_WB_SourceData/hTcells_Grk2_20230302_TErk_R1&R2/Images/0000761_01/0000761_01_TH.jpg]

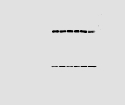

Supplement: Supplementary file 19 — Unprocessed western blot images and information file about how to open them. [file 41593_2023_1432_MOESM19_ESM.zip › F5D_WB_SourceData/hTcells_Grk2_20230302_TErk_R1&R2/Images/0000762_01/0000762_01_TH.jpg]

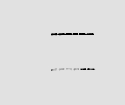

Supplement: Supplementary file 19 — Unprocessed western blot images and information file about how to open them. [file 41593_2023_1432_MOESM19_ESM.zip › F5D_WB_SourceData/hTcells_Grk2_20230302_TErk_R1&R2/Images/0000763_01/0000763_01_TH.jpg]

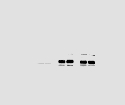

Supplement: Supplementary file 28 — Unprocessed western blot images and information file about how to open them. [file 41593_2023_1432_MOESM28_ESM.zip › S7E_WB_Images_SourceData/Images/0000503_01/0000503_01_TH.jpg]

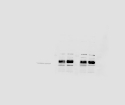

Supplement: Supplementary file 28 — Unprocessed western blot images and information file about how to open them. [file 41593_2023_1432_MOESM28_ESM.zip › S7E_WB_Images_SourceData/Images/0000504_01/0000504_01_TH.jpg]

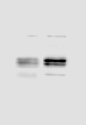

Supplement: Supplementary file 28 — Unprocessed western blot images and information file about how to open them. [file 41593_2023_1432_MOESM28_ESM.zip › S7E_WB_Images_SourceData/Images/0000504_02/0000504_02_TH.jpg]

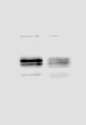

Supplement: Supplementary file 28 — Unprocessed western blot images and information file about how to open them. [file 41593_2023_1432_MOESM28_ESM.zip › S7E_WB_Images_SourceData/Images/0000504_03/0000504_03_TH.jpg]

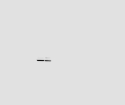

Supplement: Supplementary file 28 — Unprocessed western blot images and information file about how to open them. [file 41593_2023_1432_MOESM28_ESM.zip › S7E_WB_Images_SourceData/Images/0000505_01/0000505_01_TH.jpg]

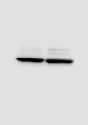

Supplement: Supplementary file 28 — Unprocessed western blot images and information file about how to open them. [file 41593_2023_1432_MOESM28_ESM.zip › S7E_WB_Images_SourceData/Images/0000505_02/0000505_02_TH.jpg]

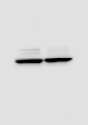

Supplement: Supplementary file 28 — Unprocessed western blot images and information file about how to open them. [file 41593_2023_1432_MOESM28_ESM.zip › S7E_WB_Images_SourceData/Images/0000505_03/0000505_03_TH.jpg]

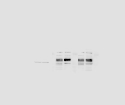

Supplement: Supplementary file 28 — Unprocessed western blot images and information file about how to open them. [file 41593_2023_1432_MOESM28_ESM.zip › S7E_WB_Images_SourceData/Images/0000506_01/0000506_01_TH.jpg]

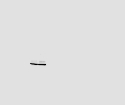

Supplement: Supplementary file 28 — Unprocessed western blot images and information file about how to open them. [file 41593_2023_1432_MOESM28_ESM.zip › S7E_WB_Images_SourceData/Images/0000507_01/0000507_01_TH.jpg]

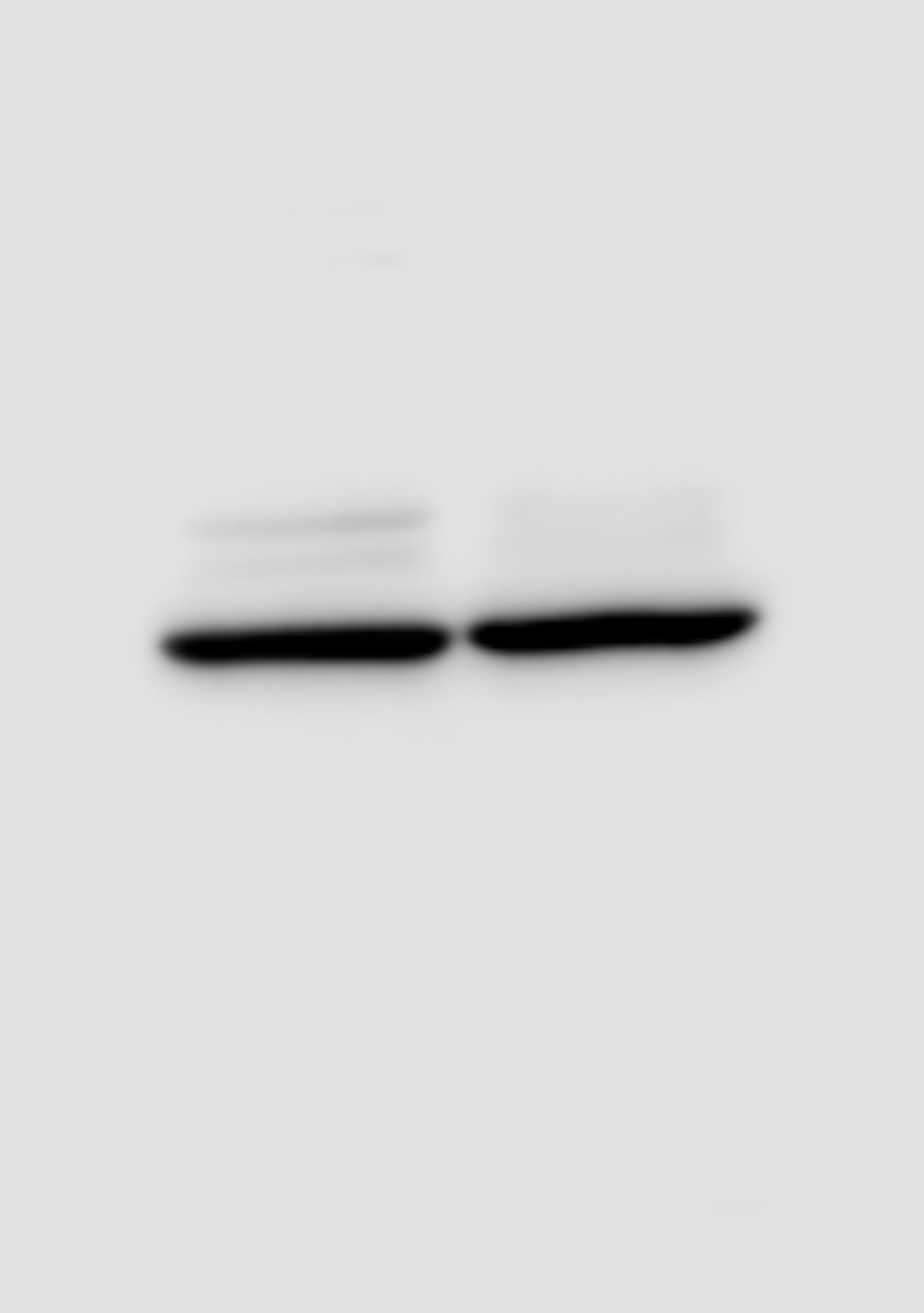

Supplement: Supplementary file 28 — Unprocessed western blot images and information file about how to open them. [file 41593_2023_1432_MOESM28_ESM.zip › S7E_WB_Images_SourceData/NT_ETS1_b-actin.png]

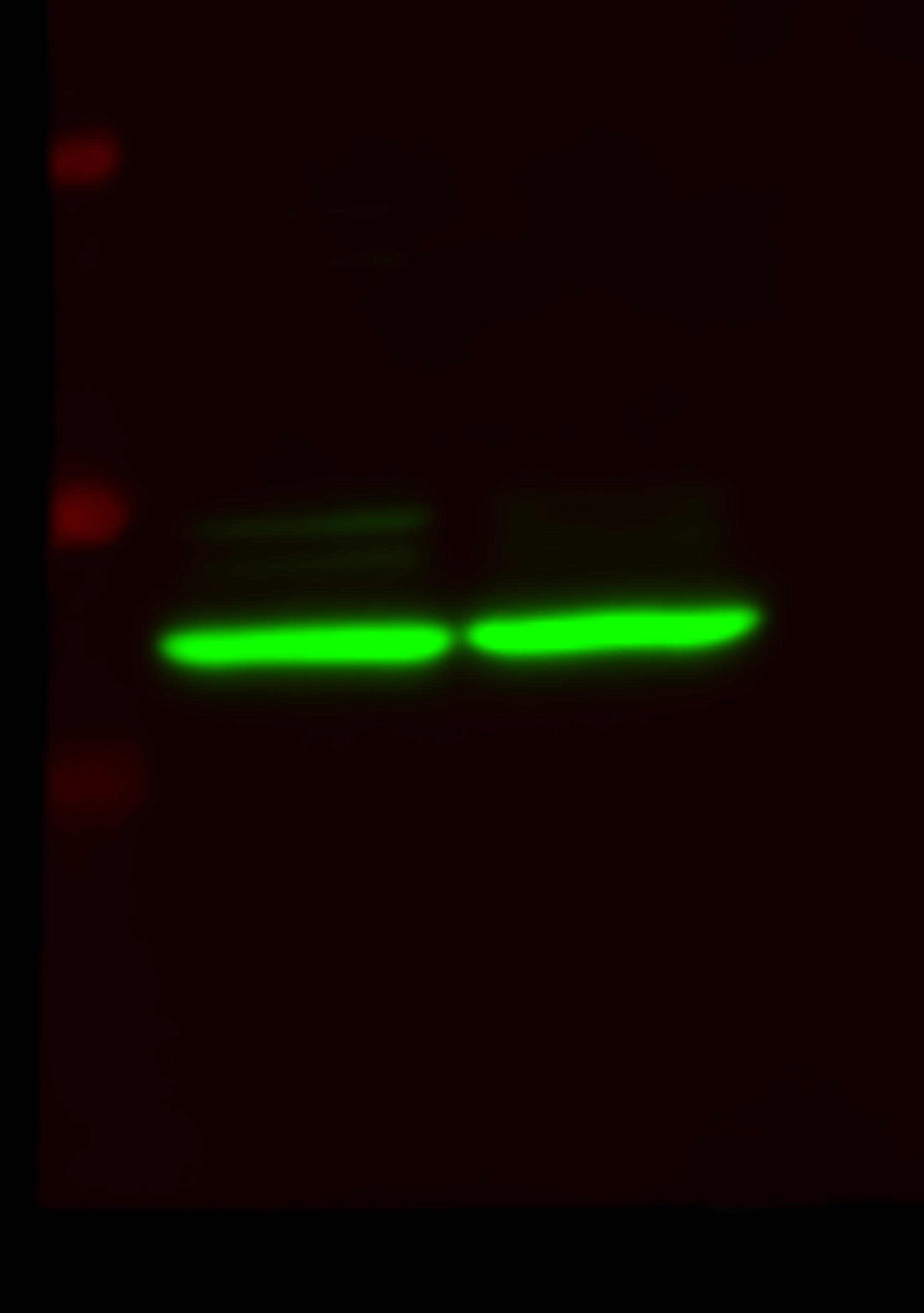

Supplement: Supplementary file 28 — Unprocessed western blot images and information file about how to open them. [file 41593_2023_1432_MOESM28_ESM.zip › S7E_WB_Images_SourceData/NT_ETS1_b-actin_ladder.png.png]

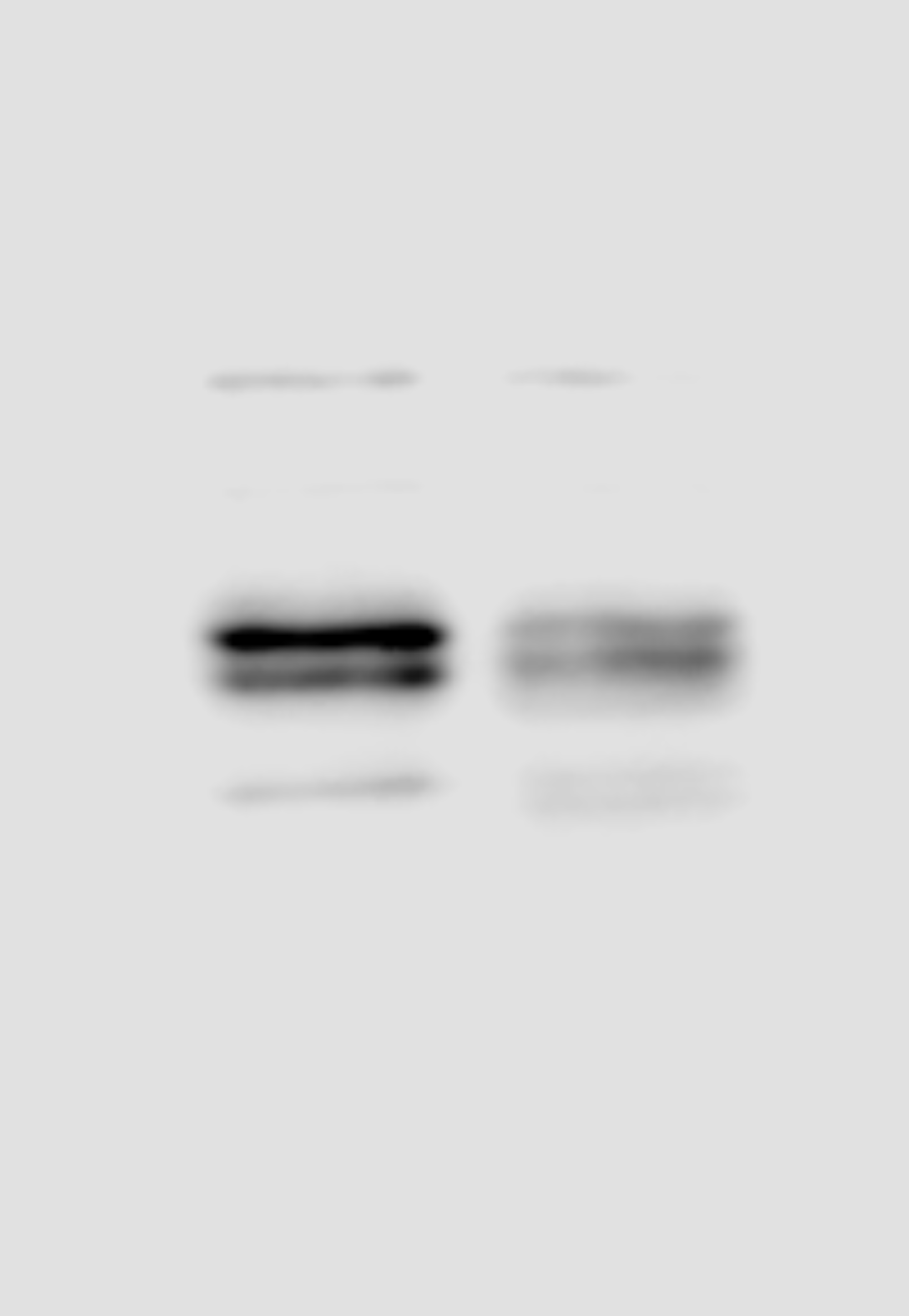

Supplement: Supplementary file 28 — Unprocessed western blot images and information file about how to open them. [file 41593_2023_1432_MOESM28_ESM.zip › S7E_WB_Images_SourceData/NT_ETS1_Ets1.png.png]

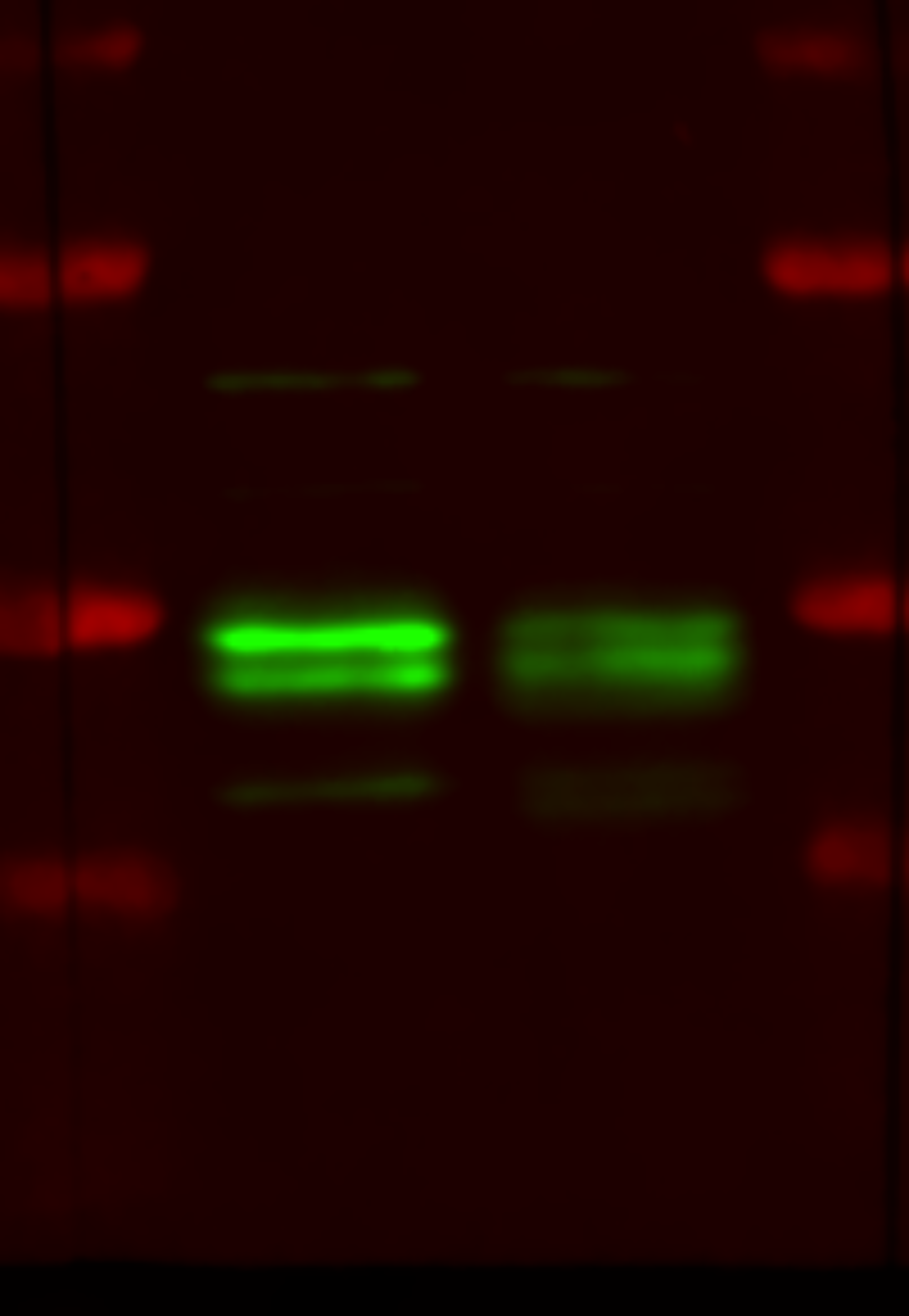

Supplement: Supplementary file 28 — Unprocessed western blot images and information file about how to open them. [file 41593_2023_1432_MOESM28_ESM.zip › S7E_WB_Images_SourceData/NT_ETS1_Ets1_ladder.png.png]
