# Supplementary material for: A genome-wide in vivo CRISPR screen identifies essential regulators of T cell migration to the CNS in a multiple sclerosis model
Source: Nat Neurosci. 2023 Sep 14;26(10):1713–25. doi: 10.1038/s41593-023-01432-2 (PMC10545543; doi:10.1038/s41593-023-01432-2)
Supplement: Supplementary file 28 — Unprocessed western blot images and information file about how to open them. [file 41593_2023_1432_MOESM28_ESM.zip › S7E_WB_Images_SourceData/NT Ets1 WB.pptx]

## Slide 1
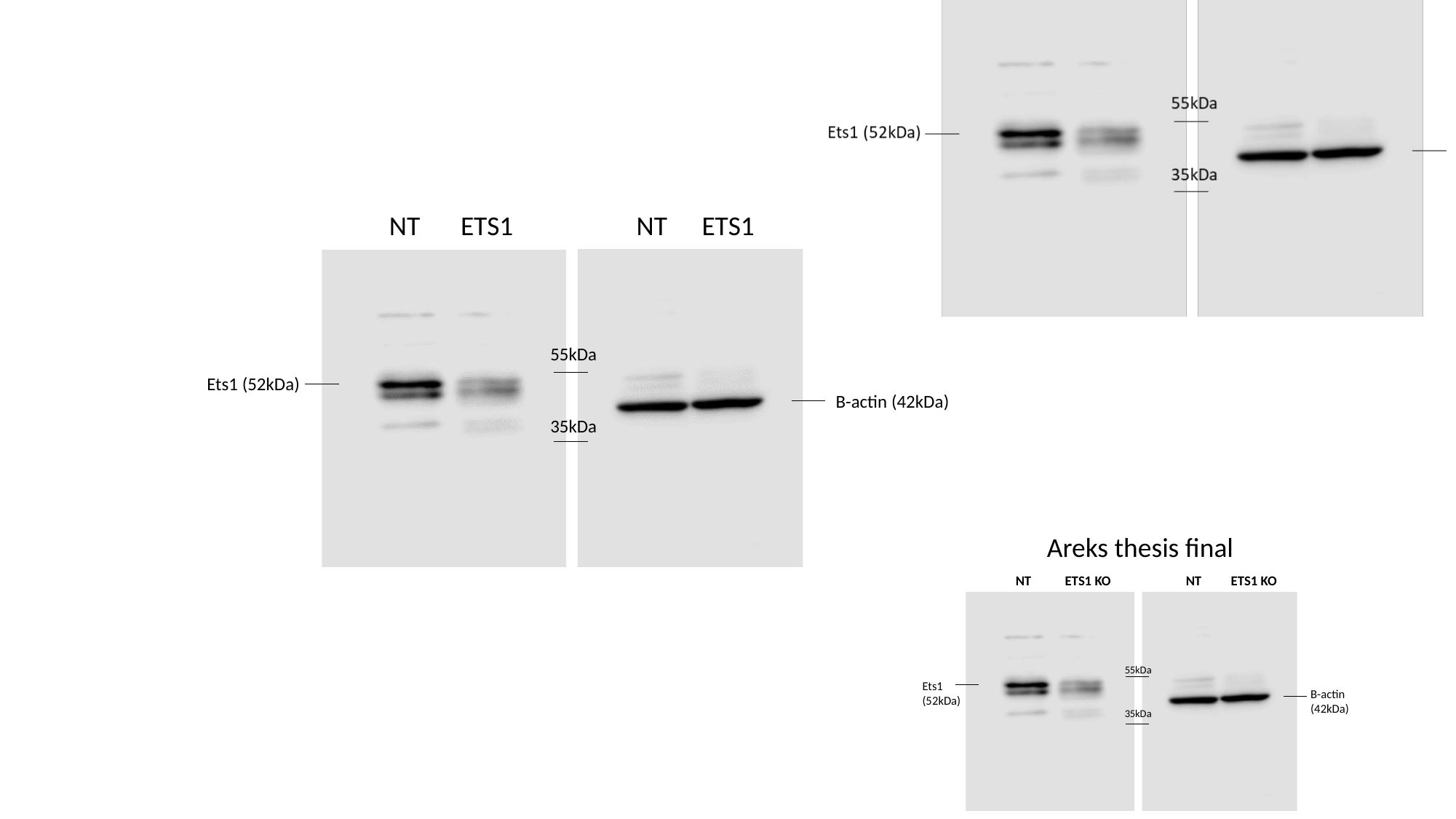

NT
ETS1
NT
ETS1
55kDa
Ets1 (52kDa)
B-actin (42kDa)
35kDa
Areks thesis final
NT
ETS1 KO
NT
ETS1 KO
55kDa
Ets1 (52kDa)
B-actin (42kDa)
35kDa
